# Exploring insecticidal activity and SAR study of newly synthesized Benzo[*h*]quinoline-based heterocycles against *Aphis craccivora* Koch. and *Culex pipiens* L. Larvae

**DOI:** 10.1038/s41598-026-48683-0

**Published:** 2026-04-24

**Authors:** Eman A. E. El-Helw, Doaa R. Abdel-Haleem, Ali Kh. Khalil, Sayed K. Ramadan

**Affiliations:** 1 Chemistry Department, Faculty of Science, Ain Shams University, Cairo, 11566 Egypt; 2 Entomology Department, Faculty of Science, Ain Shams University, Cairo, 11566 Egypt

**Keywords:** *Aphis craccivora*, Benzo[*h*]quinolines, *Culex pipiens*, Insecticidal activity, Thiazolidines, Pyrazolones, Biochemistry, Chemical biology, Chemistry, Drug discovery

## Abstract

The key material, 2-chlorobenzo[*h*]quinoline-3-carbaldehyde condensed with various active-methylene reagents to produce some benzo[*h*]quinoline-based heterocycles like pyrazolone, imidazolone, thiazolidine, pyrimidinone, and indolinone candidates. The insecticidal activities of synthesized substrates were evaluated against cowpea aphid, *Aphis craccivora*, adults and common house mosquito, *Culex pipiens* larvae. The results of toxicological bioassays demonstrated that compounds **9** and **11**, containing 2-thioxoimidazolidine and 2-thioxothiazolidine core, exhibited high insecticidal activity against *A. craccivora* adults with LC_50_s of 3.54 and 3.87 µg/mL, and against *C. pipiens* larvae with LC_50_s of 1.61 and 1.83 µg/mL, respectively. Schiff base **7** and arylidene **5** (bearing pyrazolone scaffolds) achieved good potencies with low LC_50_ values of 4.40 and 5.25 µg/mL for *A. craccivora* adults, and 2.16 and 2.49 µg/mL for *C. pipiens* larvae, respectively. On the other hand, the bulky pyrimidinetrione **16** and indolone **18** showed low toxicities. Overall, the tested substrates were more effective against *C. pipiens* larvae than *A. craccivora* adults. The current study highlighted the effect of the inserted function groups on 2-chlorobenzo[*h*]quinoline core on the compounds’ activity through the structure-activity relationship (SAR) study. This study presents promising results for the development of novel and effective insecticides to be integrated into pest control programs.

**Supplementary Information:**

The online version contains supplementary material available at 10.1038/s41598-026-48683-0.

## Introduction

The application of insecticides is the key method for limiting the outbreak or spread of harmful insects. Even so, the excessive misuse of thoughtful insecticides has harmed the ecosystem. More than five hundred insect species have developed resistance to many classes of insecticides, which cause losses of many billion dollars yearly^1,2^. Not only agricultural pests but also insect vectors have developed resistance to various synthetic chemicals^3,4^.

Cowpea aphid, *Aphis craccivora* Koch (Hemiptera: Aphididae), is a destructive pest that attacks faba beans and causes deleterious effects on them. Aphids reduce crop productivity and cause remarkable loss of plant sap, which is important for plant growth^5–8^. In addition, cowpea aphids indirectly disturb the photosynthesis process due to the formation of aphids’ honeydew secretion on leaves that promotes infection with fungus^9^. Moreover, aphids act as vectors for different plant viruses^10^. On the other hand, *Culex pipiens* L. (Diptera: Culicidae) can transmit multiple pathogens to humans and animals and contribute to the spread of diseases and recurrent outbreaks of mosquito-borne pathogens^11^. *C. pipiens* transmits multiple arboviruses, including Western and Eastern equine encephalitis viruses, Japanese encephalitis virus, and West Nile virus. Additionally, it is a vector of nematodes causing lymphatic filariasis and protists causing avian malaria^12–14^.

Hence, the increased ability of insects to build up resistance to conventional synthetic pesticides lead to an ongoing need for the exploration and development of novel insecticidal agents, particularly those that exhibit new mechanisms of action. Another main objective of identifying new chemicals is low toxicity to non-target organisms, favorable environmental profiles and cost-effective^15^.

In synthetic and pharmaceutical chemistry, nitrogen-including heterocycles are directed molecules because they serve as a fundamental platform in a diversity of biologically active chemicals^16–20^. A wide range of biological effects has been reported for quinoline (benzo[*b*]pyridine) derivatives^21–26^. The quinoline skeleton was included in some agrochemicals^27,28^. Thus, benzoquinolines have widely recognized biological efficacy, so they have been employed as scaffolds for the synthesis of many medications^29–34^. Molecules consisting of quinoline and 2-nitroimine-1,3-diazacycloalkane showed good insecticidal activity against aphids at a concentration of 500 ppm^35^. Quinolactacide is quinolone insecticide and showed good mortality against green peach aphids^36^. In turn, benzo[*h*]quinoline compounds were chosen as prospective insecticidal agents for a combination of chemical, biological, and practical (agrochemical) reasons. These reasons are well supported by experimental bioassays, molecular docking studies, and structure-activity relationship (SAR) analyses reported in the literature^25,26,29^. Several benzo[*h*]quinoline-based heterocycles exhibited significant larvicidal activity against *C. pipiens*, which in some cases their activity exceeded that of the commercial insecticide chlorpyrifos^29^.

On the other hand, a wide range of pyrazoles^37–40^, imidazoles^41–43^, and thiazolidines^44–46^ has been reported to exhibit promising pharmacological properties. Motivated by these diverse biological activities and our ongoing research interest^47–57^, we aimed to exploit benzo[*h*]quinoline aldehyde **1** as a key scaffold for the construction of hybrid systems by coupling it with various heterocyclic moieties. This strategy was achieved through its interaction with a series of active methylene compounds, with the objective of enhancing the insecticidal potential of the resulting frameworks.

### Rationale and design

The development of new insecticidal agents remains an urgent priority due to the rising resistance of agricultural pests and disease-transmitting vectors to conventional pesticides. Heterocyclic compounds, particularly those incorporating fused aromatic systems, have long been recognized for their diverse biological activities and their ability to interact effectively with enzymatic and receptor targets in insects. In this context, the benzo[*h*]quinoline scaffold offers an attractive platform for structural modification due to its chemical stability, ease of functionalization, and previously reported bioactivity profiles.

The results of the current study highlighted the significant impact of structural features on insecticidal potency within a newly synthesized series of 2-chlorobenzo[*h*]quinoline-based derivatives. The superior activity observed for sulfur-containing heterocycles such as imidazolidinone and thiazolidinone derivatives (compounds **9** and **11**) underscores the importance of sulfur atoms in enhancing lipophilicity, electronic effects, and potential interactions with insect physiological targets. Similarly, the enhanced toxicity of phenyl-substituted pyrazolone derivatives (compounds **5** and **7**) suggests that aromaticity at specific positions may facilitate improved biological recognition or membrane penetration.

Conversely, bulkier analogues such as compounds **16** and **18** exhibited markedly lower toxicity despite possessing traditionally potent functional groups (e.g., cyano, hydrazine). These findings indicate that excessive steric bulk may hinder their binding affinity or limit transport across insect tissues, thereby diminishing overall bioactivity. This clear SAR guided the strategic refinement of compound design toward frameworks that balance electronic contribution with steric accessibility.

**Fig. 1:**
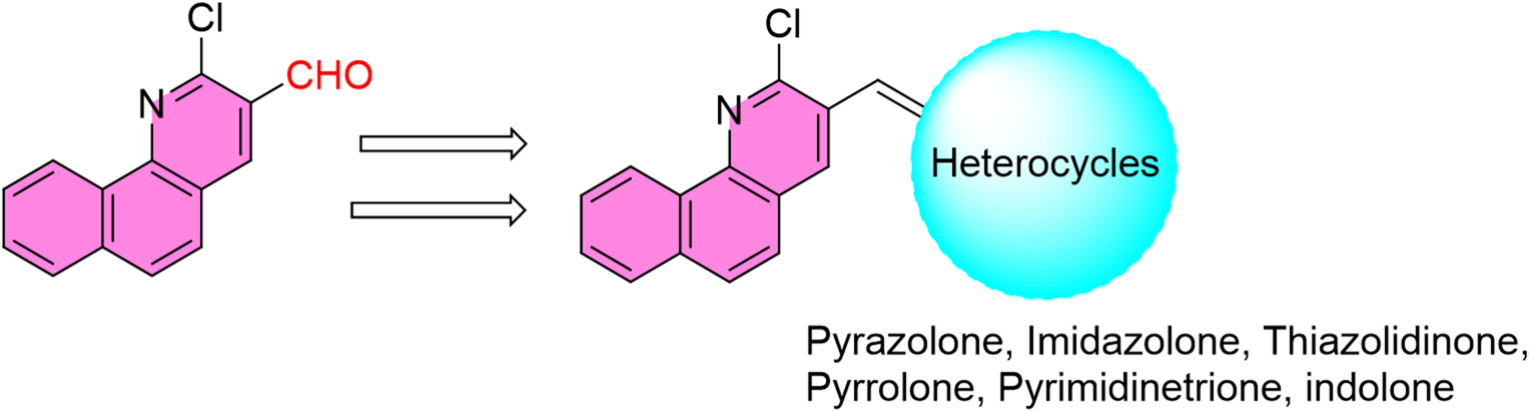
Targeted benzo[*h*]quinoline-based heterocycles.

Based on the biological outcomes and established SAR insights, the design of the compound library was guided by the following principles (cf. Fig. 1). First, the aromatic framework (2-chlorobenzo[*h*]quinoline core) was selected as the central pharmacophore due to its versatility in undergoing nucleophilic and electrophilic substitutions, its extended π-system capable of facilitating hydrophobic interactions within insect biological targets, and its documented potential in agrochemical lead discovery, Second, different heterocycles were introduced at key positions to modulate electron density, hydrogen-bonding capability, lipophilicity, and molecular flexibility. Special emphasis was placed on sulfur-containing heterocycles (imidazolidinone and thiazolidinone), informed by their robust performance and known biochemical relevance. Third, exploration of aromatic substitution was achieved through pyrazolone, imidazolone, and thiazolidinone scaffolds. For example, pyrazolone derivatives bearing a phenyl ring were included to evaluate the role of aromatic reinforcement. The strong insecticidal activity of these analogues confirmed the design hypothesis that phenyl incorporation enhances molecular recognition.

Fourth, avoidance of excessive steric bulk was taken into consideration based on the observed inactivity of bulkier compounds (**16** and **18**). The design framework prioritized moderate steric profiles, minimal crowding around key binding regions, and controlled introduction of functional groups with known insecticidal potential, such as cyano or hydrazide moieties. Fifth, emphasis on modular, simple synthetic routes were achieved. A practical synthetic design was adopted to allow rapid generation of diverse analogues, enable facile modification for SAR studies, and support potential upscaling for future biological testing. Finally, mode-of-action exploration was performed. Compounds demonstrating the highest activity were intentionally designed with functional groups amenable to molecular docking studies, biochemical assays targeting insect enzymes/proteins, and mechanistic investigations to guide future, more selective insecticide design.

While the synthetic strategies employed in this study rely on well-established condensation reactions, the novelty of the present work lies not in the individual reactions per se, but in the systematic integration of structural diversification, comparative insecticidal evaluation, and SAR analysis centered on the underexplored 2-chlorobenzo[*h*]quinoline scaffold. The introduction of diverse bioactive heterocyclic motifs (pyrazolone, imidazolone, thiazolidine, pyrimidinone, and indolinone) onto the 2-chlorobenzo[*h*]quinoline core and their parallel evaluation against two agriculturally and medically important insect species (*A. craccivora* adults and *C. pipiens* larvae) has not been previously reported. The study goes beyond routine synthesis by offering a head-to-head comparison of multiple functional groups within a unified molecular framework, allowing clear correlations between structural features and insecticidal efficacy to be established.

Importantly, the results reveal distinct and SAR trends, particularly the pronounced activity of compounds incorporating 2-thioxoimidazolidine and 2-thioxothiazolidine moieties, which exhibited LC₅₀ values comparable to, or better than, several reported synthetic insecticides. In contrast, sterically demanding motifs (pyrimidinetrione and indolinone) resulted in marked activity loss, providing meaningful mechanistic insight into the role of steric and electronic effects. The consistent observation that the synthesized compounds are more potent against *C. pipiens* larvae than *A. craccivora* adults further underscores the biological relevance of the scaffold and supports its selectivity profile. Thus, the novelty of this work resides in the rational functionalization of a rarely exploited benzo[*h*]quinoline aldehyde precursor, the comparative biological assessment of structurally diverse heterocycles within a single chemical series, and the derivation of clear SAR conclusions that can guide future insecticide design. Taken together, these aspects elevate the study provide new chemical–biological insight relevant to the development of next-generation insecticidal agents suitable for integrated pest management programs.

## Results and discussion

### Chemistry

**Scheme 1:**
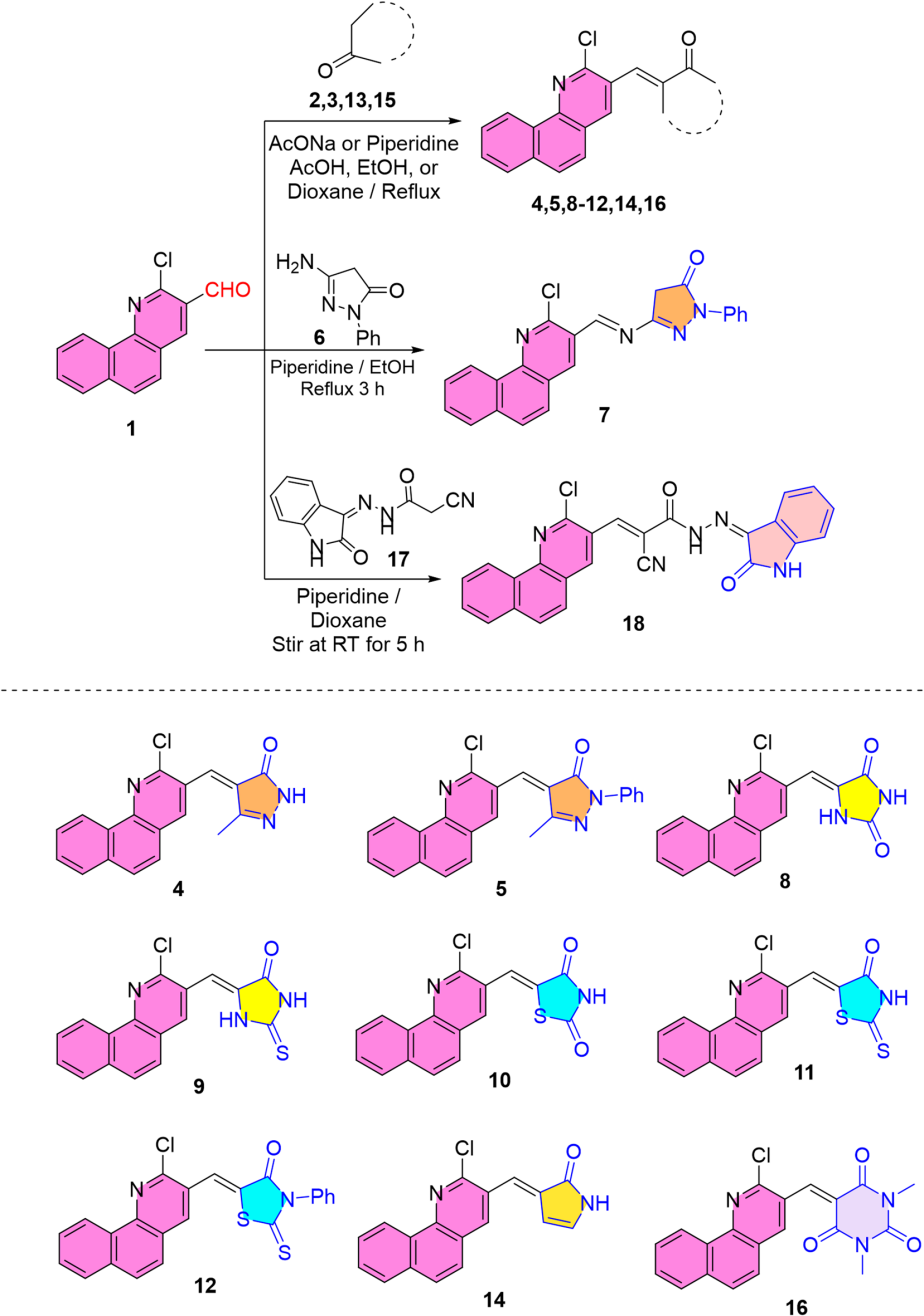
Condensation of benzoquinoline aldehyde **1** (scaffold colored in purple) with various active methylene-bearing heterocyclic reagents.

Substrates incorporating a benzoquinoline framework are biologically significant due to their well-documented insecticidal properties, which arise from the scaffold’s extended π-conjugation and its ability to engage effectively in enzyme and receptor interactions^25,26,29^. Thus, the key aldehyde functionality in 2-chlorobenzo[*h*]quinoline-3-carbaldehyde **(1)**^58^ was utilized for the synthesis of a variety of heterocycles based on a benzo[*h*]quinoline core (colored in purple) (cf. Scheme 1). First, condensation of aldehyde **1** with pyrazolone derivatives **2** and **3** in refluxing anhydrous sodium acetate / glacial ethanoic acid and piperidine / ethyl alcohol afforded arylidene candidates **4** and **5**^31^, respectively (cf. Scheme 1). Their IR spectra lacked aldehyde functionality and showed amide-carbonyl absorptions. A singlet signal for methyl protons appeared in their ^1^H NMR spectra in the upfield region. Also, a broad singlet signal of amide NH proton was detected in the downfield region in ^1^H NMR spectrum of pyrazolone **4**. Otherwise, treating aldehyde **1** with 3-amino-1-phenylpyrazol-5-one **(6)** furnished Schiff base derivative **7** (cf. Scheme 1). Its ^1^H NMR spectrum retained a singlet signal for methylene (CH_2_) protons, showed a singlet signal for methine proton (CH = N), and lacked primary amino protons signal.

In turn, imidazolidinone and thiazolidinone derivatives exhibited remarkable insecticidal potency, positioning them as promising scaffolds for the development of next-generation agrochemical agents. Thus, gathering both benzoquinoline and imidazolidinones / thiazolidinones in one framework was achieved through the condensation of aldehyde **1** with imidazolidinones and thiazolidinones (cf. Scheme 1). Initially, treatment of aldehyde **1** with imidazolidine-2,4-dione and its 2-thioxo analog in refluxing ethyl alcohol / anhydrous sodium acetate and dioxane / piperidine produced arylidenes **8** and **9**, respectively. For arylidene **8**, the vibrational coupling of imide carbonyl groups was detected in its IR spectrum. For arylidene **9**, absorption bands for NH, C = O, and C = S groups appeared in its IR spectrum. Their ^1^H NMR spectra revealed two broad singlet signals for two NH protons in the downfield region. Likewise, treating aldehyde **1** with thiazolidin-2,4-dione, 2-thioxothiazolidin-4-one, and 3-phenyl-2-thioxothiazolidin-4-one in refluxing glacial ethanoic acid including sodium acetate anhydrous afforded arylidenes **10–12**^59,60^, respectively (cf. Scheme 1). The vibrational coupling of carbonyl functionalities appeared in IR spectrum of arylidene **10**. For arylidenes **10** and **11**, their ^1^H NMR charts disclosed a broad singlet signal for NH proton in the downfield region, which was not present in arylidene **12**.

Treating aldehyde **1** with pyrrolin-2-one **13** in refluxing dioxane involving anhydrous sodium acetate offered the arylidene **14** (Scheme 1). Its IR and ^1^H NMR spectra displayed absorption band and broad singlet signal for amide NH, respectively. Treatment of aldehyde **1** with 1,3-dimethylpyrimidine-2,4,6(1*H*,3*H*,5*H*)-trione (**15**) in 1,4-dioxane and piperidine at room temperature remained unchanged for long time. So, the reaction was carried out under refluxing conditions to produce benzoquinolyl-pyrimidinetrione **16** (Scheme 1). Its IR spectrum showed absorption bands for carbonyl groups with vibrational coupling. Also, its ^1^H NMR spectrum illustrated singlet signal for two methyl protons. Lastly, condensation of aldehyde **1** with the active methylene of isatin cyanoacetohydrazone **17** upon stirring in dioxane and piperidine at an ambient temperature produced the α,β-unsaturated nitrile candidate **18** (Scheme 1). Its IR spectrum revealed absorption bands for carbonyl and NH functionalities and demonstrated the decreasing in frequency of stretching absorption of nitrile group due to new conjugation with C = C linkage. Furthermore, its ^1^H NMR spectrum provided two broad singlet signals for two NH protons in the downfield region. The reaction times, solvents and bases used, and yields of compounds obtained were displayed in Table 1.

**Table 1:** Reaction conditions and yields of the synthesized compounds.*.

Compds.	Reaction Solvent/Base	Reaction time (h)	Yield (%)
**4**	AcOH/AcONa	4	78
**5**	EtOH/Piperidine	5	74
**7**	EtOH/Piperidine	3	71
**8**	EtOH/AcONa	7	79
**9**	Dioxane/Piperidine	6	83
**10**	AcOH/AcONa	5	78
**11**	AcOH/AcONa	6	85
**12**	AcOH/AcONa	5	81
**14**	Dioxane/AcONa	6	63
**16**	Dioxane/Piperidine	8	72
**18**	Dioxane/Piperidine	5	69

*All reactions were carried out under reflux conditions, except for compound 18 was obtained at room temperature.

### Insecticidal activity

The insecticidal potency of benzo[*h*]quinoline-based heterocycles was screened against agricultural and public health pests, *A. craccivora* and *C. pipiens*, respectively. The synthesized compounds were evaluated by two different methods of exposure against *A. craccivora* adults and *C. pipiens* Larvae.

### Adulticidal activity for Aphis craccivora

From Table 2, some benzo[*h*]quinoline-based compounds exhibited good adulticidal potency against *A. craccivora* adults in comparison to imidacloprid. The most active substrates were **9**,** 11**,** 7**, and **5** with LC_50_ of 3.54, 3.87, 4.40 and 5.25 µg/mL, respectively, relative to imidacloprid (3.69 µg/mL). On the other hand, compounds **4**, **16**, **14**, and **18** showed least effectiveness with high LC_50_ values, 21.22, 18.21, 16.35, and 12.03 µg/mL, respectively. The insertion of different moieties affects the activity, where compounds **9**, **11**, **7**, and **5** were highly active 5.99, 5.48, 4.82, and 4.04 times than compound **4**. While compounds **16**, **14**, and **18** were less potent and only 1.16, 1.29, and 1.76 times relative to compound **4**. At the same time, moderate activities were shown by compounds **12**, **8**, and **10** with potency 3.41, 2.71, and 2.18 folds than compound **4**. The population showed good homogeneity for most tested compounds except compound **4** (7.75).

**Table 2:** Adulticidal activity for novel benzo[*h*]quinoline-based heterocycles against *Aphis craccivora* adults, 48 h post-treatment.

Compds.	LC_25_ (µg/mL) (^a^F.l. at 95%)	LC_50_ (µg/mL) (^a^F.l. at 95%)	LC_90_ (µg/mL) (^a^F.l. at 95%)	^b^Slope ± SE	^c^X^2^	^d^ *P*	Relative potency
**4**	6.94 (5.51–8.45)	21.22 (17.38–26.89)	177.45 (114.41- 114.41)	1.38 ± 0.12	7.75	0.10	1
**5**	1.71 (1.19–2.25)	5.25 (4.27–6.32)	44.07 (32.37–66.57)	1.38 ± 0.12	2.97	0.56	4.04
**7**	1.51 (1.06–1.99)	4.40 (3.58–5.28)	33.36 (25.48–47.38)	1.45 ± 0.12	4.36	0.35	4.82
**8**	2.34 (1.66–3.05)	7.82 (6.40–9.50)	77.21 (53.19- 128.59)	1.28 ± 0.14	0.67	0.95	2.71
**9**	1.26 (0.86–1.67)	3.54 (2.84–4.28)	25.21 (19.42–25.21)	1.50 ± 0.12	3.70	0.44	5.99
**10**	2.78 (1.99–3.60)	9.72 (7.95–11.94)	104.51 (69.09- 185.72)	1.24 ± 0.13	0.88	0.92	2.18
**11**	1.31 (0.90–1.74)	3.87 (3.10–4.67)	30.25 (23.43–42.02)	1.43 ± 0.11	2.60	0.62	5.48
**12**	1.89 (1.31–2.50)	6.22 (5.06–7.54)	59.50 (42.06–95.33)	1.30 ± 0.15	0.81	0.93	3.41
**14**	5.00 (3.84–6.21)	16.35 (13.41–20.49)	155.10 (99.95- 285.67)	1.31 ± 0.17	2.15	0.70	1.29
**16**	5.87 (4.61–7.19)	18.21 (14.99–22.80)	156.32 (102.13–281.40)	1.37 ± 0.12	4.99	0.28	1.16
**18**	3.58 (2.65–4.53)	12.03 (9.90- 14.83)	120.17 (120.17- 214.09)	1.28 ± 0.14	0.97	0.91	1.76
**Imidacloprid**	1.21 (0.81–1.64)	3.69 (2.93–4.50)	30.53 (23.42–43.07)	1.39 ± 0.16	1.00	0.90	5.75

^a^ (F.l.) Fiducial limits.

^b^Slope of the concentration-inhibition regression line ± standard error.

^c^X^2^ chi-square significant at *P* < 0.05.

^d^P probability.

### Larvicidal activity for Culex pipiens

All tested compounds showed high activity relative to temephos (WHO recommended larvicide), which used as reference larvicide. The compounds **9**, **11**, **7**, and **5** achieved high efficiency with low LC_50_ of 1.61, 1.83, 2.16, and 2.49 µg/mL, respectively, in comparison with other tested compounds and temephos. These compounds were highly potent with 30.76, 27.06, 22.93, and 19.89 folds than temephos, respectively (cf. Table 3). Similarly to adulticidal activity against *A. craccivora*, **4** (9.74 µg/mL), **16** (8.22 µg/mL), **14** (6.87 µg/mL), and **18** (5.33 µg/mL) exhibited low activity relative to other synthesized compounds. But these compounds showed relative effectiveness 5.08, 6.02, 7.20, and 9.29 times than temephos (Table 3). The chi-square values indicated the homogeneous response of *C. pipiens* larvae population.

**Table 3:** Larvicidal activity for novel benzo[*h*]quinoline derivatives against *Culex pipiens* larvae, 48 h post-treatment.

Compds.	LC_25_ (µg/mL) (^a^F.l. at 95%)	LC_50_ (µg/mL) (^a^F.l. at 95%)	LC_90_ (µg/mL) (^a^F.l. at 95%)	^b^Slope ± SE	^c^X^2^	^d^ *P*	Relative potency
**4**	3.10 (2.43–3.79)	9.74 (7.98–12.31)	85.87 (55.20- 158.68)	1.35 ± 0.12	5.95	0.20	5.08
**5**	0.74 (0.49–1.01)	2.49 (1.99–3.04)	24.67 (17.59–39.10)	1.28 ± 0.16	0.74	0.94	19.89
**7**	0.65 (0.42–0.89)	2.16 (1.70–2.65)	21.15 (15.27–32.95)	1.29 ± 0.18	1.35	0.85	22.93
**8**	1.12 (0.79–1.47)	3.74 (3.06–4.53)	36.41 (25.26–59.98)	1.29 ± 0.14	1.05	0.90	13.24
**9**	0.50 (0.31–0.71)	1.61 (1.24–1.99)	14.48 (10.72–21.79)	1.34 ± 0.12	1.10	0.89	30.76
**10**	1.29 (0.91–1.67)	4.42 (3.62–5.40)	45.86 (30.87–79.16)	1.26 ± 0.13	1.04	0.90	11.20
**11**	0.51 (0.31–0.74)	1.83 (1.39–2.29)	20.25 (14.21–33.30)	1.22 ± 0.12	0.72	0.94	27.06
**12**	0.85 (0.57–1.15)	3.02 (2.42–3.70)	33.27 (22.85–55.93)	1.23 ± 0.11	0.87	0.92	16.40
**14**	1.97 (1.46–2.50)	6.87 (5.61–8.59)	73.44 (46.79- 138.07)	1.24 ± 0.14	2.48	0.64	7.20
**16**	2.46 (1.88–3.07)	8.22 (6.72–10.35)	81.22 (51.73- 152.53)	1.28 ± 0.17	3.66	0.45	6.02
**18**	1.55 (1.12–1.99)	5.33 (4.37–6.55)	55.57 (36.67–98.88)	1.26 ± 0.13	2.28	0.68	9.29
**Temephos**	17.20 (13.92–20.74)	49.53 (40.57–62.99)	369.25 (240.32- 669.81)	1.46 ± 0.13	6.06	0.19	1

^a^(F.l.) Fiducial limits.

^b^Slope of the concentration-inhibition regression line ± standard error.

^c^X^2^ chi-square significant at *P* < 0.05.

^d^P probability.

## Discussion

The synthesized compounds were evaluated against agricultural pest (*A. craccivora*) and disease vector (*C. pipiens*) to generalize their application against two insect species with different hazards. The tested compounds were applied by two methods of exposure to insect pests, the contact method for *C. pipiens* larvae, to evaluate their penetration rate through the larval cuticle. To assess their contact and systematic activity, they were used by the leaf dipping method against *A. craccivora* adults. The obtained result demonstrated that the *C. pipiens* larvae were more susceptible to the tested compounds than *A. craccivora* adults. The compounds enter the *C. pipiens* larval body through the cuticle by contact with the skin and the hydrophobicity of the tested compounds increases their penetration rate, therefore achieve high activity. The low solubility of compounds in water decreases translocation in plants, hence their systemic activity decreases as noted in treatment of *A. craccivora* adults^61–64^.

Benzo[*h*]quinoline-bearing heterocycles are promising as insecticidal agents against many insect species. The isolated benzylisoquinoline alkaloids from various plant species were evaluated for toxicity against the fruit fly and codling moth, and these compounds demonstrated both chronic and acute insecticidal effectiveness. The most substantial effects were the deformations, feeding alteration, and death effects. In addition, these compounds may target the ecdysone and octopamine receptor as a possible mechanism of action^65^. 4,7-Dichloroquinoline derivative showed remarkable larvicidal and pupicidal efficacy against dengue and malaria vectors^66^. 7-Chloro-4-(1*H*-1,2,3-triazol-1-yl)quinoline derivatives exhibited antifeedant and insecticidal activities against *Spodoptera frugiperda* (J.E. Smith) at concentrations ranging from 250 to 1000 µg/mL^67^.

The insertion of different moieties enhanced insecticidal activities. A series of pyrazole derivatives exhibited notable efficiency against a broad spectrum of insect pests, including *Helicoverpa armigera*, *A. craccivora*, *C. pipiens pallens*^68^, and 2nd and 4th larval instars *S. frugiperda*, showing good interaction with the GABA receptor^69^. Some phenylpyrazoles bearing 1-methoxyaryl or arylimine groups had insecticidal potency against *A. craccivora*, *C. pipiens pallens*, *Plutella xylostella* and *Mythimna separata*. Those compounds had higher larvicidal activity against *C. pipiens* than aphicidal activity. Insecticides containing phenylpyrazole were successful in public hygiene and crop protection by targeting glutamate-gated chloride (GluCl) channels and gamma-aminobutyric acid (GABA)-gated chloride channels^70^. In addition, phenylpyrazole containing chlorophenyl, two cyano moieties and a pyrazole ring showed high efficiency against second and fourth instar of *Spodoptera littoralis*^71^.

In addition to good insecticidal activity of compounds containing the thiazolidine moiety against *A. craccivora*, they had low toxicity to bees. Electrophysiological studies indicated that those compounds targeted insect nAChR, which were verified by the docking and quantum mechanics calculation^72,73^. Also, this moiety was effective against *S. frugiperda* and *Bemisia tabaci*^74^. Compounds bearing pyrrolone exhibit insecticidal potency through disrupting mitochondrial function, specifically uncoupling oxidative phosphorylation and inhibiting ATP synthesis.

### Structure-activity relationship (SAR)

The construction of bioactive hybrid molecules with two or more moieties significantly enhances insecticidal activity and delays the development of resistance. The synthesized structures, besides 2-chlorobenzo[*h*]quinoline, have different moieties inserted instead of 3-carbaldehyde. The insertion of 2-thioxoimidazolidine and 2-thioxothiazolidine in compounds **9** and **11**, respectively, greatly improved their toxicity against both *A. craccivora* adults and *C. pipiens* larvae. Notably, the presence of sulfur atoms in the imidazolidine and thiazolidine rings significantly elevated the insecticidal potency, as shown in compounds **9** and **11**.

Moreover, a thioamide group of arylidene **9** showed high efficacy compared to oxygen-containing compound **8**, suggesting that sulfur substitution is responsible for cuticle permeability or target affinity^75^. Compound **11** with two sulfur atoms also exhibited high potency compared to **10** with two oxygen atoms. Although compound **12** bears a similar dithio-moiety, it showed reduced activity, likely due to the bulky phenyl substituent, which lowers bioavailability. Compounds **8** and **10**, which have no or fewer sulfur substitutions, demonstrated lower potency. Overall, these findings reinforce the importance of sulfur in improving potency as well as the minimal bulky groups that favor higher insecticidal effects (Scheme 1)^76^.

The presence of a phenyl group attached to the pyrazolone ring in compounds **7** and **5** greatly elevated the insecticidal efficiency relative to arylidene **4**; this group may cause a hydrophobic interaction with the target site^77^. Schiff base **7** demonstrated higher insecticidal effectiveness than arylidene **5** against the tested insects. Possibly, the presence of amino linker connecting the phenyl pyrazolone substitution to 2-chlorobenzo[*h*]quinoline ring is mainly responsible for the significant activity of Schiff base **7**. This amino linkage may influence molecular flexibility, which increases binding affinity with the insect target site^78,79^.

Also, Scheme 1 highlights that the compounds **14**, **18**, and **16** are bulky and rigid substituents on the 2-chlorobenzo[*h*]quinoline core, which reduce insecticidal potency. Compound **14** is a 1,3-dihydropyrrol-2-one derivative; the direct fusion of the pyrrolone group to the core ring likely provides structural rigidity but limited flexibility, resulting in low binding affinity. Despite the presence of insecticidal active cyano and hydrazide groups in indolone **18**, the bulking decreases the activity, due to repulsion with the target site^80,81^. Compound **16** bears dimethyl groups on the pyrimidin-2,4,6-(1*H*,3*H*,5*H*)-trione, contributing to steric hindrance and reducing its activity.

Overall, these findings provide valuable insight for designing more effective insecticidal agents by optimizing linker presence and substitution patterns. Also, these insights suggest that minimizing steric bulk and enhancing molecular flexibility could improve insecticidal potency in this chemical class.

## Conclusion

In this study, a straightforward and efficient synthetic strategy enabled the preparation of a diverse series of 2-chlorobenzo[*h*]quinoline-bearing heterocycles. The biological evaluation of these compounds against *A. craccivora* adults and *C. pipiens* L. larvae revealed notable differences in insecticidal potency that were clearly influenced by structural variations. Among the synthesized compounds, the sulfur-containing imidazolidinone and thiazolidinone derivatives (**9** and **11**) demonstrated the most pronounced insecticidal activity, exhibiting remarkably low LC_50_ values. This highlights the significance of sulfur-based heterocyclic frameworks in enhancing bioactivity within this chemical series. Similarly, pyrazolone derivatives incorporating a phenyl moiety, particularly compounds **5** and **7**, showed strong insecticidal effectiveness, suggesting that aromatic substitution at this scaffold may play a critical role in promoting interaction with biological targets. In contrast, bulkier derivatives such as compounds **16** and **18** displayed noticeably reduced toxicity, despite containing functional groups traditionally associated with insecticidal efficacy, including cyano and hydrazine groups. Their diminished performance may be attributed to steric hindrance that impedes optimal binding affinity or reduces their ability to penetrate insect tissues.

Overall, the results establish a clear SAR within this newly synthesized compound series, underscoring how subtle structural modifications can dramatically influence insecticidal behavior. These findings not only expand the chemical space of potential insecticidal agents but also lay the groundwork for further optimization. Given the promising activity observed for selected derivatives, future studies should focus on elucidating their precise modes of action, assessing their selectivity toward target pests, and evaluating their environmental safety profiles. Such investigations will be essential for advancing these compounds toward practical application and for guiding the rational design of next-generation, more selective insecticides.

## Materials and methods

Melting points were measured on a MEL-TEMP II electric melting point apparatus (Thomas Scientific, NJ, USA). The FT-IR spectra were measured using potassium bromide (KBr) disks on a Fourier transform infrared Thermo Electron Nicolet 7600 spectrometer (Thermo Fisher Scientific Inc., MA, USA) at Faculty of Science, Ain Shams University. The ^1^H NMR spectra were run at 300 *MHz* on GEMINI NMR spectrometer (GEMINI, Manufacturing & Engineering Inc., CA, USA) operating tetramethyl silane (TMS) as an internal standard in DMSO‑*d*_6_ (deuterated dimethyl sulfoxide) at Faculty of Science, Cairo University. The EI-MS (electron impact mass spectra) were recorded on a Shimadzu GC-MS-QP-1000EX mass spectrometer applying at 70 eV (Shimadzu Scientific Instruments, Inc., MD, USA). Elemental analyses were recorded using a Perkin-Elmer 2400 CHN elemental analyzer (Waltham, MA) at Faculty of Science, Ain Shams University, and satisfactory analytical data (± 0.4) were obtained for all compounds. The progress of reactions and purity of compounds obtained were monitored and checked by thin layer chromatography (TLC) applying Merck Kiesel gel 60F_254_ analytical sheets (Merck, Fluka, Switzerland) using appropriate mobile phase (diethyl ether and ethyl acetate). All chemical structures were drawn by ChemDraw, version 22.2.0.3300 (Revvity Signals Software, Cambridge, MA, USA; https://revvitysignals.com/products/research/chemdraw).

### (E/Z)-4-((2-Chlorobenzo[h]quinolin-3-yl)methylene)-5-methyl-2,4-dihydro-3H-pyrazol-3-one (4)

A mixture of benzo[*h*]quinoline-aldehyde **1** (1 mmol) and 5-methyl-2,4-dihydropyrazol-3-one **(2)** (1 mmol) in glacial ethanoic acid (15 mL) including anhydrous sodium acetate (1 mmol) was refluxed for 4 h. The solid obtained was filtered and recrystallized by 1,4-dioxane to furnish brown crystals, mp. 290–292 °C, yield 78%. FT-IR (KBr, ν, cm^− 1^): 3202 (NH), 1648 (C = O), 1610 (C = N). ^1^H NMR (300 *MHz*, DMSO-*d*_6_, δ, ppm): 2.63 (s, 3H, CH_3_), 7.62–8.05 (m, 6H, Ar-H), 8.86 (s, 1H, CH=), 9.20 (s, 1H, CH benzoquinoline), 12.23 (*br*.s, 1H, NH). EI-MS (70 eV, *m/z*, %): 321.68 (M^+^^.^, 23). Anal. Calcd. for C_18_H_12_ClN_3_O (321.7640): C, 67.19; H, 3.76; N, 13.06; Found: C, 67.11; H, 3.71; N, 13.05%.

### (E/Z)-4-((2-Chlorobenzo[h]quinolin-3-yl)methylene)-5-methyl-2-phenyl-2,4-dihydro-3H-pyrazol-3-one (5)^31^

A mixture of aldehyde **1** (1 mmol) and 5-methyl-2-phenyl-2,4-dihydropyrazol-3-one **(3)** (1 mmol) in absolute ethanol (15 mL) containing piperidine (0.1 mL) was refluxed for 5 h. The precipitated solid while heating was filtered and recrystallized by 1,4-dioxane to acquire beige crystals, mp. 324–326 °C [Lit. mp. 322–324 °C], yield 74%.

### (E/Z)-5-(((2-Chlorobenzo[h]quinolin-3-yl)methylene)amino)-2-phenyl-2,4-dihydro-3H-pyrazol-3-one (7)

A mixture of aldehyde **1** (1 mmol) and 5-amino-2-phenyl-2,4-dihydropyrazol-3-one **(6)** (1 mmol) in absolute ethanol (15 mL) containing piperidine (0.1 mL) was refluxed for 3 h. The solid separated during heating was collected and crystallized from 1,4-dioxane to acquire beige crystals, mp. 315–317 °C, yield 71%. FT-IR (KBr, ν, cm^− 1^): 1656 (C = O), 1621 (C = N). ^1^H NMR (300 *MHz*, DMSO-*d*_6_, δ, ppm): 4.00 (s, 2H, CH_2_), 7.15–8.01 (m, 9H, Ar-H), 8.04 (s, 1H, CH = N), 8.36 (d, 1H, CH benzoquinoline, *J* = 8.4 *Hz*), 8.70 (d, 1H, CH benzoquinoline, *J* = 8.1 *Hz*), 8.90 (s, 1H, CH benzoquinoline). EI-MS (70 eV, *m/z*, %): 398.76 (M^+^^.^, 28). Anal. Calcd. for C_23_H_15_ClN_4_O (398.8500): C, 69.26; H, 3.79; N, 14.05; Found: C, 69.19; H, 3.72; N, 14.03%.

### (E/Z)-5-((2-Chlorobenzo[h]quinolin-3-yl)methylene)imidazolidine-2,4-dione (8)

A mixture of aldehyde **1** (1 mmol) and imidazolidine-2,4-dione (1 mmol) in absolute ethyl alcohol (15 mL) containing anhydrous sodium acetate (1 mmol) was refluxed for 7 h. The solid separated on hot was collected and crystallized from 1,4-dioxane to acquire beige crystals, mp. 320–322 °C (decomp.), yield 79%. FT-IR (KBr, ν, cm^− 1^): 3200, 3158 (NH), 1766, 1712 (C = O vibrational coupling), 1656 (C = N). ^1^H NMR (300 *MHz*, DMSO-*d*_6_, δ, ppm): 7.55 (s, 1H, CH=), 7.78–8.07 (m, 6H, Ar-H), 8.60 (s, 1H, CH benzoquinoline), 10.43 (*br*.s, 1H, NH), 10.68 (*br*.s, 1H, CONHCO). EI-MS (70 eV, *m/z*, %): 323.59 (M^+^^.^, 34). Anal. Calcd. for C_17_H_10_ClN_3_O_2_ (323.7360): C, 63.07; H, 3.11; N, 12.98; Found: C, 62.99; H, 3.07; N, 12.96%.

### (E/Z)-5-((2-Chlorobenzo[h]quinolin-3-yl)methylene)-2-thioxoimidazolidin-4-one (9)

A mixture of aldehyde **1** (1 mmol) and 2-thioxoimidazolidine-4-one (1 mmol) in 1,4-dioxane (15 mL) containing piperidine (0.1 mL) was refluxed for 6 h. The solid separated after cooling was collected and crystallized from ethanol/dioxane mixture (2:1) to acquire beige crystals, mp. 294–296 °C (decomp.), yield 83%. FT-IR (KBr, ν, cm^− 1^): 3464, 3346 (NH), 1685 (C = O), 1364 (C = S). ^1^H NMR (300 *MHz*, DMSO-*d*_6_, δ, ppm): 7.76–7.98 (m, 6H, Ar-H), 8.72 (s, 1H, CH=), 8.88 (s, 1H, CH benzoquinoline), 10.33 (*br*.s, 1H, NHCS), 11.54 (*br*.s, 1H, CONHCS). EI-MS (70 eV, *m/z*, %): 339.68 (M^+^^.^, 24). Anal. Calcd. for C_17_H_10_ClN_3_OS (339.7970): C, 60.09; H, 2.97; N, 12.37; Found: C, 60.00; H, 2.93; N, 12.35%.

### Reaction of aldehyde 1 with thiazolidinone derivatives

A mixture of aldehyde **1** (1 mmol) and thiazolidine-2,4-dione, 2-thioxothiazolidine-4-one, or 3-phenyl-2-thioxothiazolidine-4-one (1 mmol) in glacial ethanoic acid (15 mL) containing anhydrous sodium acetate (1 mmol) was refluxed for 5–6 h (followed by TLC). The solid separated was collected and crystallized from 1,4-dioxane to furnish arylidenes **10–12**, respectively.

### (E/Z)-5-((2-Chlorobenzo[h]quinolin-3-yl)methylene)thiazolidine-2,4-dione (10)^59^

Orange crystals, mp. >360 °C [Lit. mp. >360 °C], yield 78%.

### (E/Z)-5-((2-Chlorobenzo[h]quinolin-3-yl)methylene)-2-thioxothiazolidin-4-one (11)^59^

Brown crystals, mp. 310–312 °C (decomp.) [Lit. mp. 299–301 °C], yield 85%.

### (E/Z)-5-((2-Chlorobenzo[h]quinolin-3-yl)methylene)-3-phenyl-2-thioxothiazolidin-4-one (12)^60^

Yellow crystals, mp. 312–314 °C [Lit. mp. 305–307 °C], yield 81%.

### (E/Z)-3-((2-Chlorobenzo[h]quinolin-3-yl)methylene)-1,3-dihydro-2 H-pyrrol-2-one (14)

A mixture of aldehyde **1** (1 mmol) and 1,3-dihydropyrrol-2-one **(13)** (1 mmol) in 1,4-dioxane (15 mL) including anhydrous sodium acetate (1 mmol) was refluxed for 6 h. After cooling, the solid obtained was filtered and crystallized by ethanol/dioxane mixture (2:1) to furnish beige crystals, mp. 260–262 °C, yield 63%. FT-IR (KBr, ν, cm^− 1^): 3349 (NH), 1685 (C = O), 1620 (C = N). ^1^H NMR (300 *MHz*, DMSO-*d*_6_, δ, ppm): 7.82–8.11 (m, 8H, Ar-H), 8.93 (s, 1H, CH=), 9.02 (s, 1H, CH benzoquinoline), 10.42 (*br*.s, 1H, NH). EI-MS (70 eV, *m/z*, %): 306.64 (M^+^^.^, 19). Anal. Calcd. for C_18_H_11_ClN_2_O (306.7490): C, 70.48; H, 3.61; N, 9.13%; Found: C, 70.40; H, 3.56; N, 9.12%.

### 5-((2-Chlorobenzo[h]quinolin-3-yl)methylene)-1,3-dimethylpyrimidine-2,4,6(1 H,3 H,5 H)-trione (16)

A mixture of aldehyde **1** (1 mmol) and 1,3-dimethylbarbituric acid (**15**) (1 mmol) in 1,4-dioxane (15 mL) including piperidine (0.2 mL) was refluxed for 8 h. During heating, the precipitated solid was collected and recrystallized by 1,4-dioxane to get beige crystals, mp. 306–308 °C, yield 72%. FT-IR (KBr, ν, cm^− 1^): 1700, 1664 (C = O vibrational coupling), 1630 (C = N). ^1^H NMR (300 *MHz*, DMSO-*d*_6_, δ, ppm): 3.52 (s, 6H, 2 CH_3_), 7.72–8.17 (m, 6H, Ar-H), 8.27 (s, 1H, CH=), 8.94 (s, 1H, CH benzoquinoline). EI-MS (70 eV, *m/z*, %): 379.72 (M^+^^.^, 35). Anal. Calcd. for C_20_H_14_ClN_3_O_3_ (379.8000): C, 63.25; H, 3.72; N, 11.06; Found: C, 63.16; H, 3.67; N, 11.03%.

### (E/Z)-3-(2-Chlorobenzo[h]quinolin-3-yl)-2-cyano-N’-((Z)-2-oxoindolin-3-ylidene)acrylohydrazide (18)

A mixture of aldehyde **1** (1 mmol) and 2-cyano-*N*’-(2-oxoindolin-3-ylidene)acetohydrazide (**17**) (1 mmol) in 1,4-dioxane (15 mL) including piperidine (0.2 mL) was stirred at room temperature for 5 h (followed by TLC). The color of solution was changed into red and the solid obtained was collected by filtration and recrystallized by 1,4-dioxane to produce red crystals, mp. 222–224 °C, yield 69%. FT-IR (KBr, ν, cm^− 1^): 3175 (NH), 2210 (C ≡ N), 1729 (C = O indolone), 1708 (C = O), 1617 (C = N). ^1^H NMR (300 *MHz*, DMSO-*d*_6_, δ, ppm): 5.88 (s, 1H, CH=), 6.72–8.14 (m, 10H, Ar-H), 8.77 (s, 1H, CH benzoquinoline), 9.14 (*br*.s, 1H, NH), 10.59 (*br*.s, 1H, NH indolone). EI-MS (70 eV, *m/z*, %): 451.72 (M^+^^.^, 27). Anal. Calcd. for C_25_H_14_ClN_5_O_2_ (451.8700): C, 66.45; H, 3.12; N, 15.50; Found: C, 66.39; H, 3.09; N, 15.51%.

### Biological evaluation

#### Insects rearing

##### Aphis craccivora

The lab strain of the *A. craccivora* adults was provided by the Central Agricultural Pesticides Laboratory, Giza, Egypt, which was maintained under regulated conditions, 22 ± 2 °C with relative humidity 70–80% and a 10–14 h (light: dark) photoperiod, without exposure to any insecticides. The insects were housed on faba beans that were grown in small pots with a diameter of 20 cm. The pots of the faba bean were maintained in controlled conditions until needed^82^.

##### Culex pipiens

Lab strain of third instar larvae *C. pipiens* were provided by the insectary at Entomology Department, Faculty of Science, Ain Shams University, which were reared under controlled conditions at 25 ± 3 °C with relative humidity, 75–85% and a 10–14 h (light: dark) photoperiod and away from exposure to any chemicals^83^. The egg rafts were collected daily in glass containers, and the hatched larvae were fed on fish feed till pupation. The pupae were transferred to adult cages for emergence. Sucrose solution (10%) was prepared for the adults^84^.

##### Toxicological activity

Samples of *A. craccivora* adults and *C. pipiens* larvae were brought to the Toxicology laboratory for the bioassays of synthesized compounds. The synthesized compounds were dissolved in DMSO to obtain stock solutions and subsequently diluted in distilled water to perform six serial dilutions.

##### Adulticidal activity for Aphis craccivora

The adulticidal activity of the tested compounds was assessed on *A. craccivora* adults using the leaf dipping technique^85^. The faba bean leaves were cut into 25 mm leaf discs, then immersed for 15 s in the prepared serial dilutions (1.56, 3.125, 6.25, 12.5, 25, 50 µg/mL) of each tested compound and Imidacloprid (Best^®^ 20% WP, El Helb Co., Egypt). To dry the discs, they were placed upside down on agar in the bottom of small plastic cups (30 mm) for 30 min. For the negative control group, leaf discs were immersed in DMSO (1%). Six replicates for each concentration, each with ten apterous adults. Mortality% was calculated 48 h post-treatment.

##### Larvicidal activity for Culex pipiens

Twenty larvae of *C. pipiens* were added to 60 mL of each solution concentration (0.78, 1.56, 3.12, 6.25, 12.50, and 25 µg/mL). DMSO (1%) and Temephos (LARVIGUARD^®^, 50% EC), Shri Ram Agro Chemicals, India, with concentrations of 100, 50, 25, 12.50, 6.25, and 3.12 µg/mL, were used as negative and positive controls, respectively. All treatments were replicated three times. Mortality% was calculated 48 h post-treatment^86,87^.

### Statical analysis

A total of sixty apterous adults, *A. craccivora*, were used in a bioassay test, six replicates for each concentration, each with 10 apterous adults. Also, sixty third instar *C. pipiens*, three replicates were performed for each concentration, twenty larvae per replicate. The control mortalities were less than 5% so they were not corrected. The mortalities were collected after 48 h. Probit regression data were determined for *A. craccivora* and *C. pipiens* from calculated mortality percentages according to Finney (1971)^88^, using Logarithmic dose probit line software (Ldp line, Ehab software, Egypt) (https://www.ehabsoft.com/ldpline/). To compare the activities of synthesized compounds, relative potencies were calculated^89^.

## Supplementary Information

Below is the link to the electronic supplementary material.

Supplementary Material 1

## Data Availability

All data generated in this study can be found in the supplementary information files.
