# Supplementary material for: Exploring insecticidal activity and SAR study of newly synthesized Benzo[h]quinoline-based heterocycles against Aphis craccivora Koch. and Culex pipiens L. Larvae
Source: Sci Rep. 2026 Apr 24;16:13401. doi: 10.1038/s41598-026-48683-0 (PMC13109357; doi:10.1038/s41598-026-48683-0)
Supplement: Supplementary file 1 — Supplementary Material 1 [file 41598_2026_48683_MOESM1_ESM.pdf]

Supporting information:

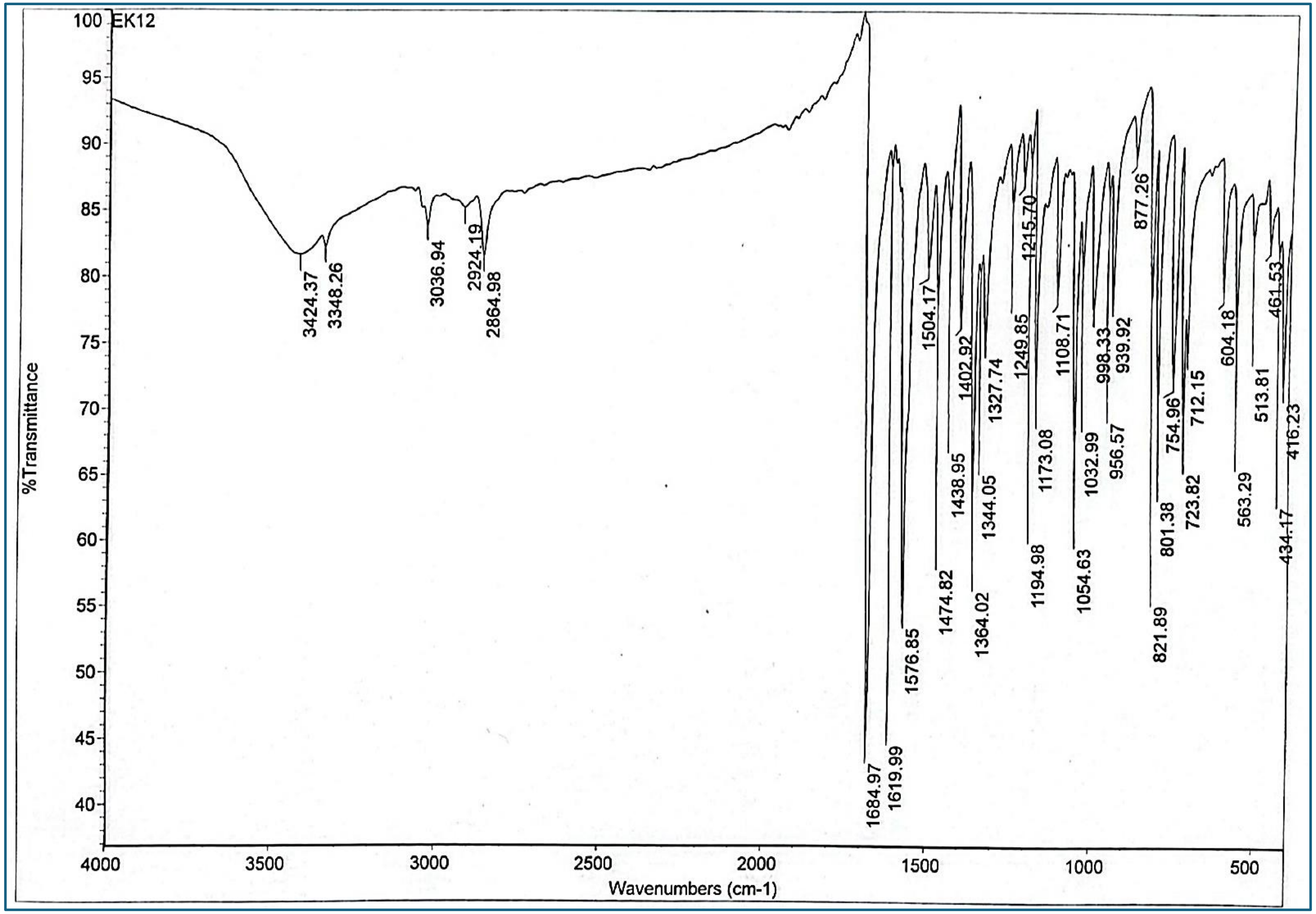

IR spectrum of compound 1

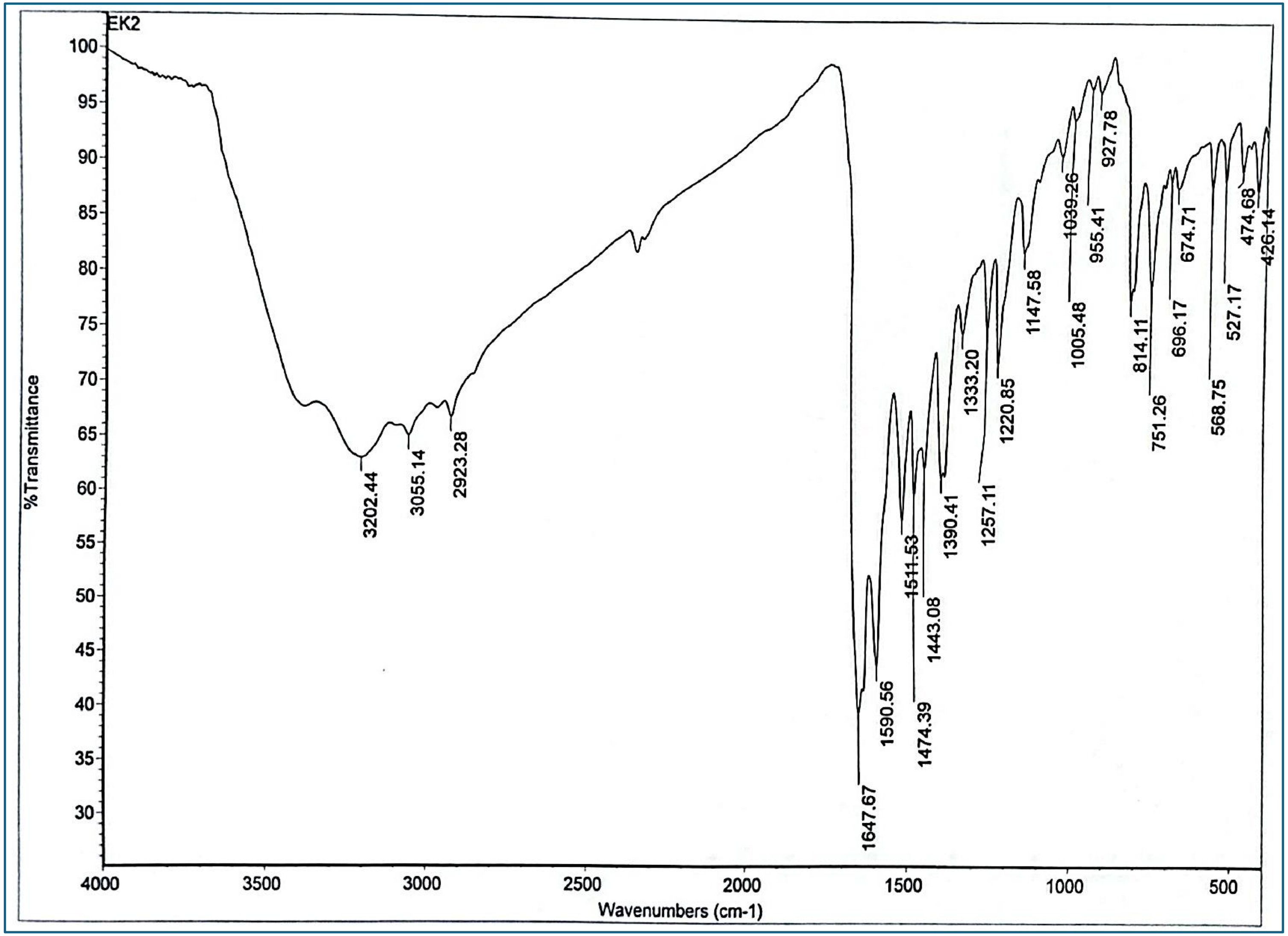

IR spectrum of compound 4

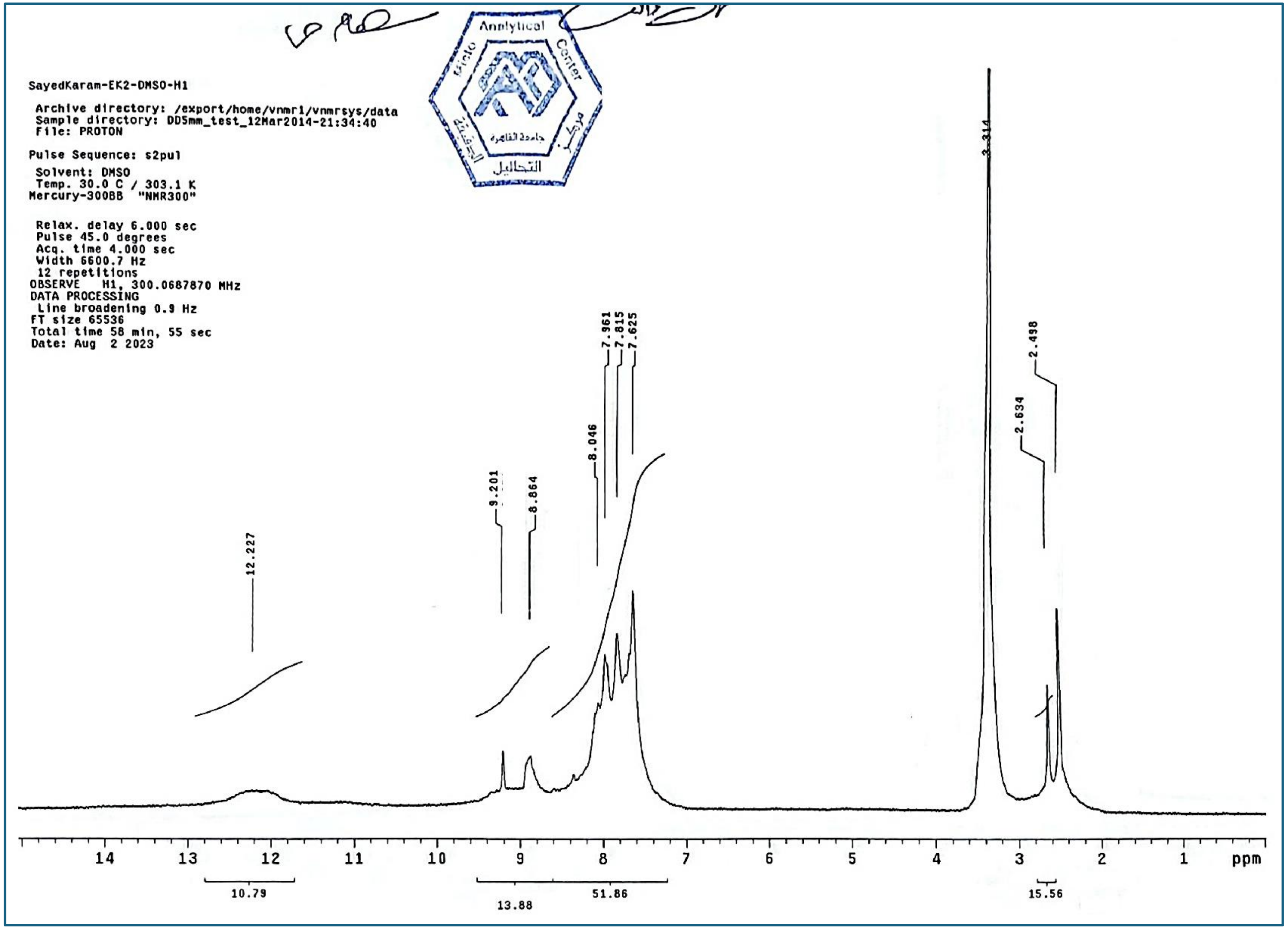

^{1}H NMR spectrum (DMSO-d_6) of compound 4

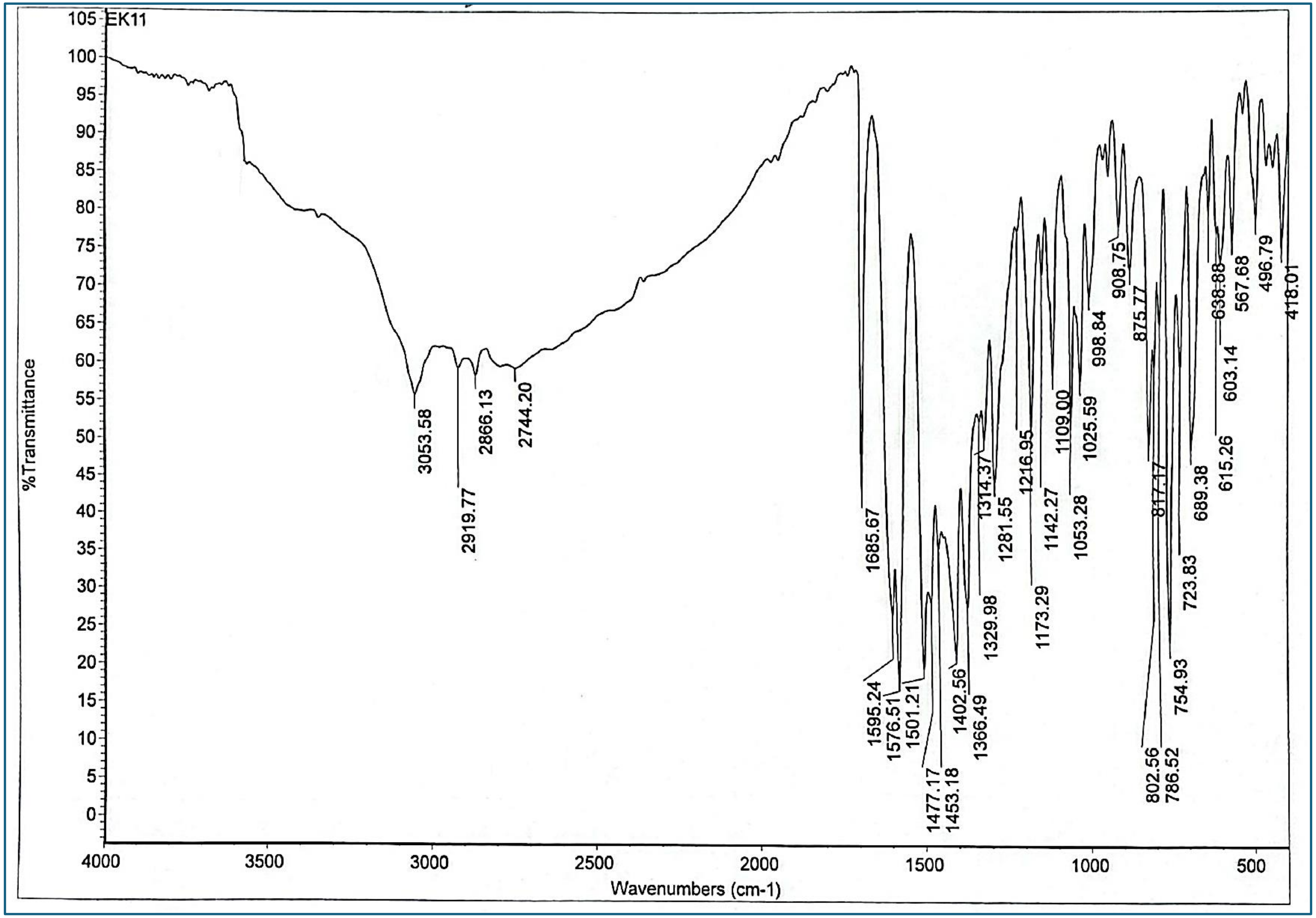

IR spectrum of compound 5

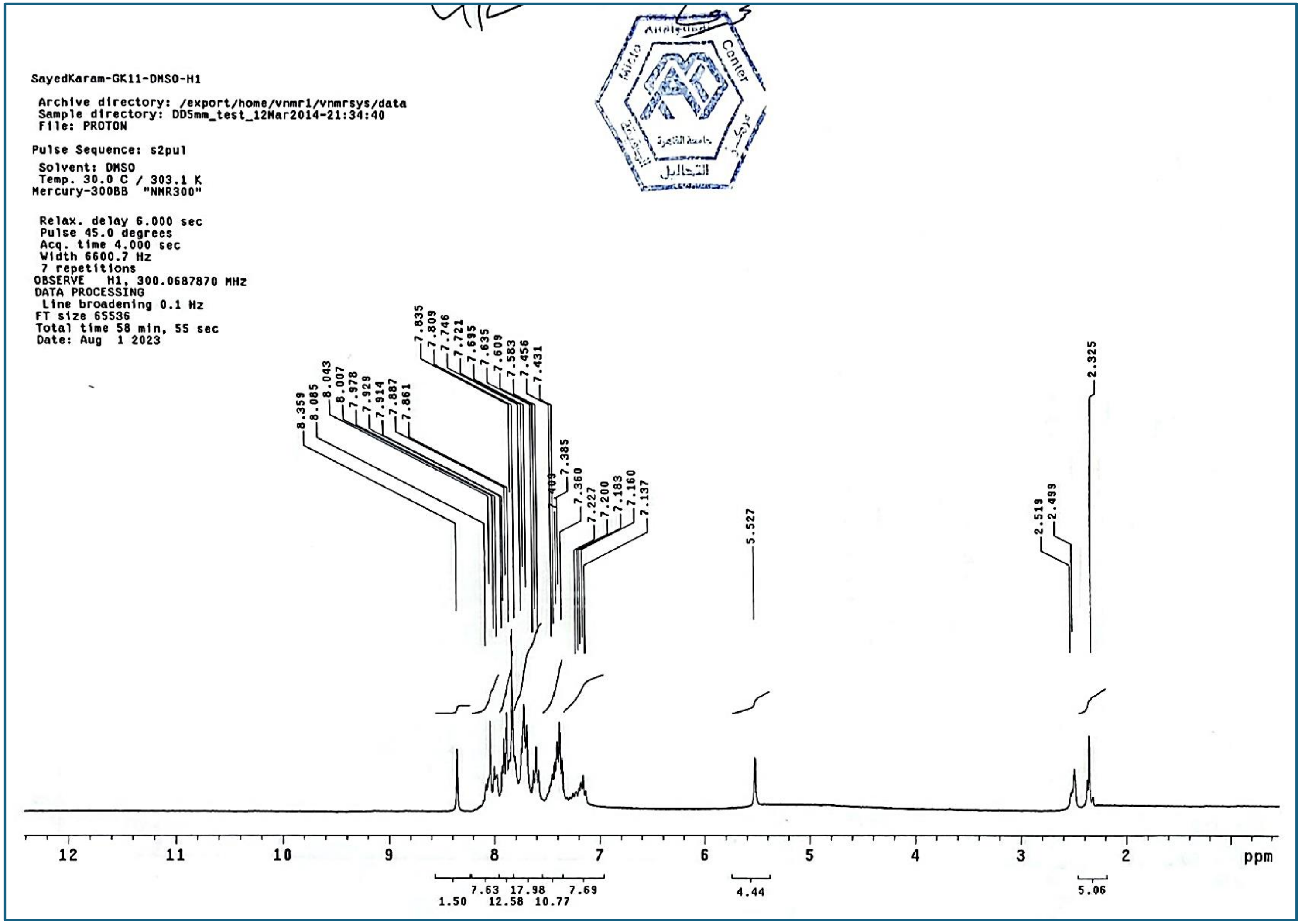

^{1}H NMR spectrum (DMSO-d_6) of compound 5

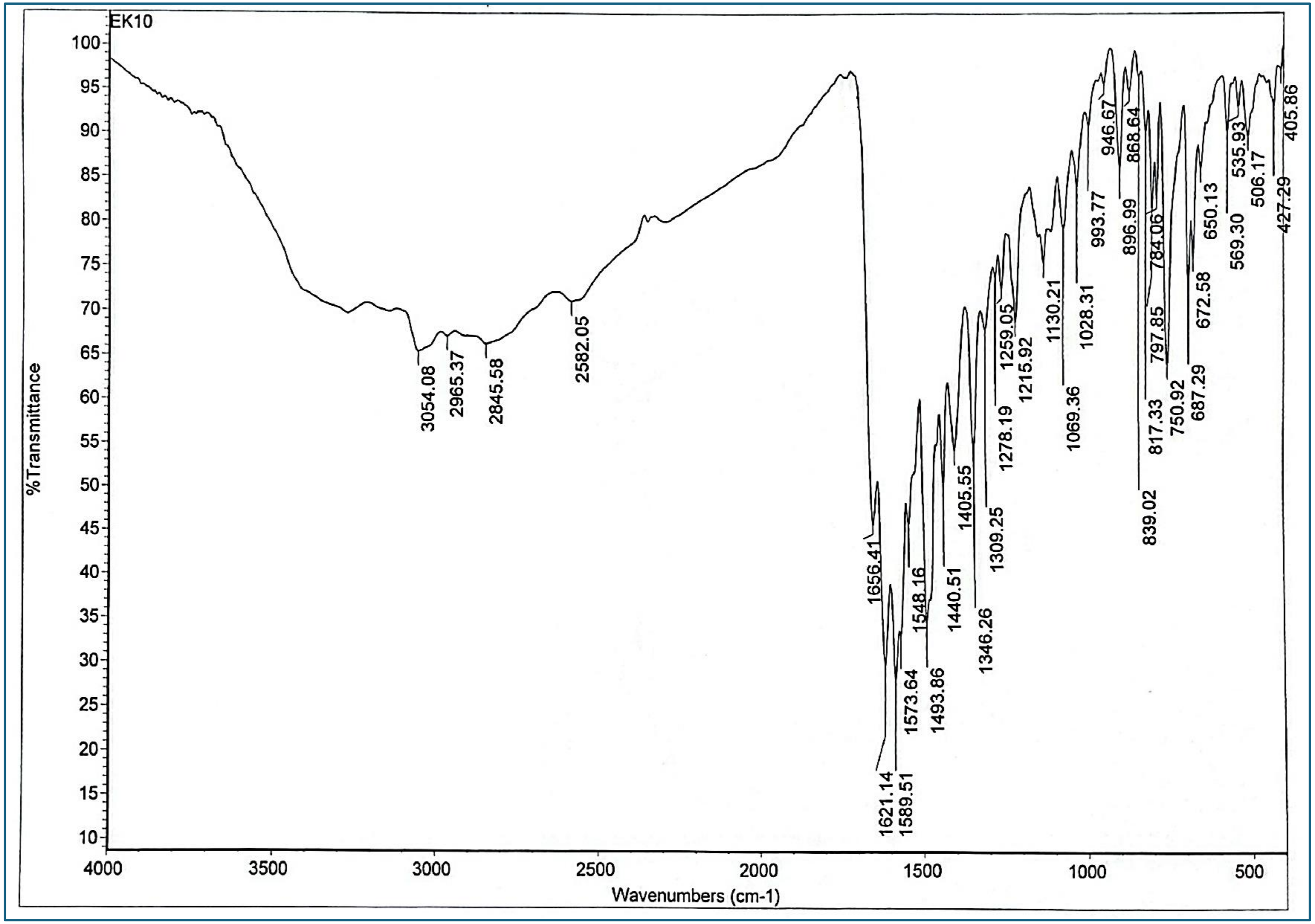

IR spectrum of compound 7

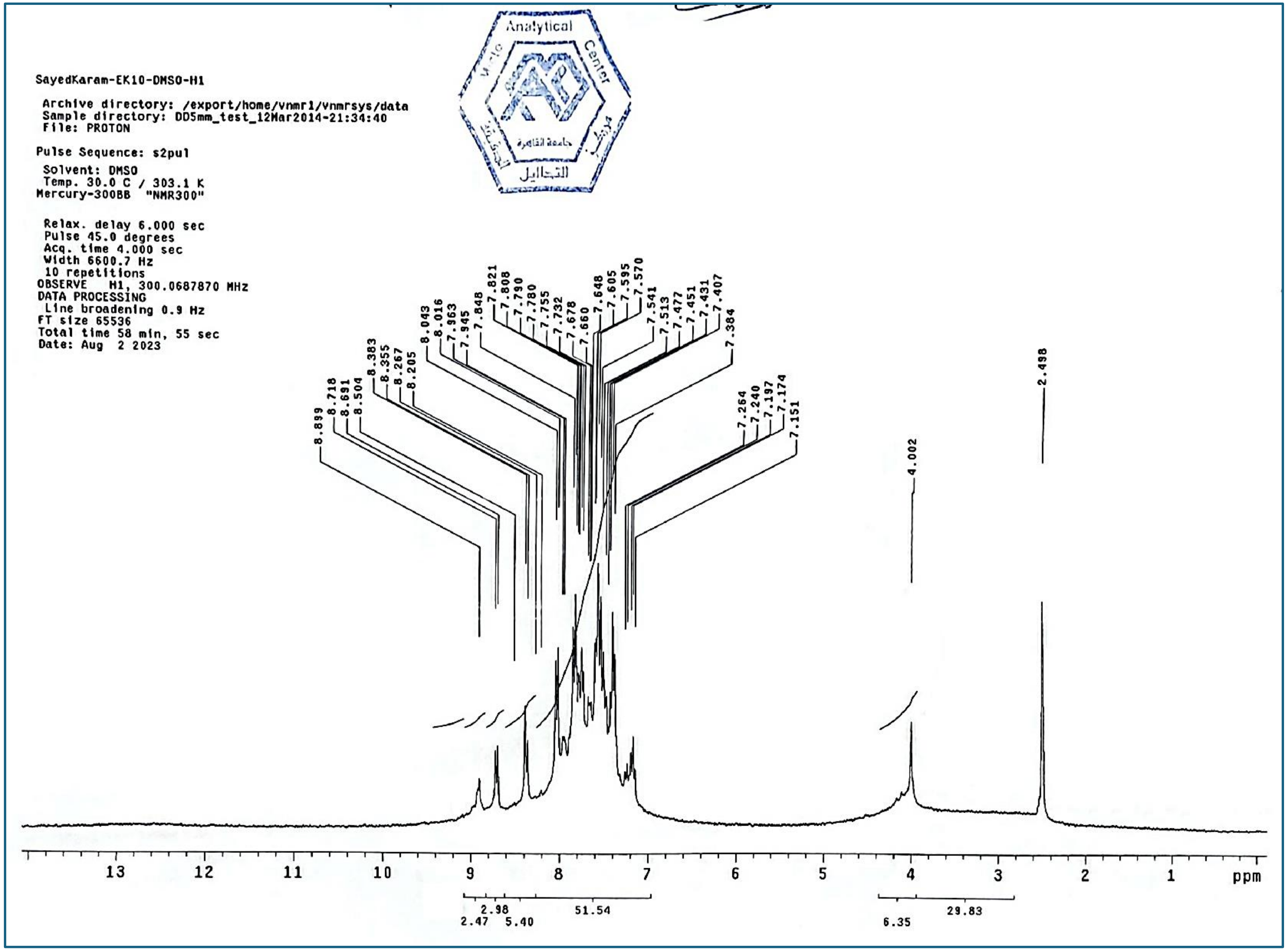

^{1}H NMR spectrum (DMSO-d_6) of compound 7

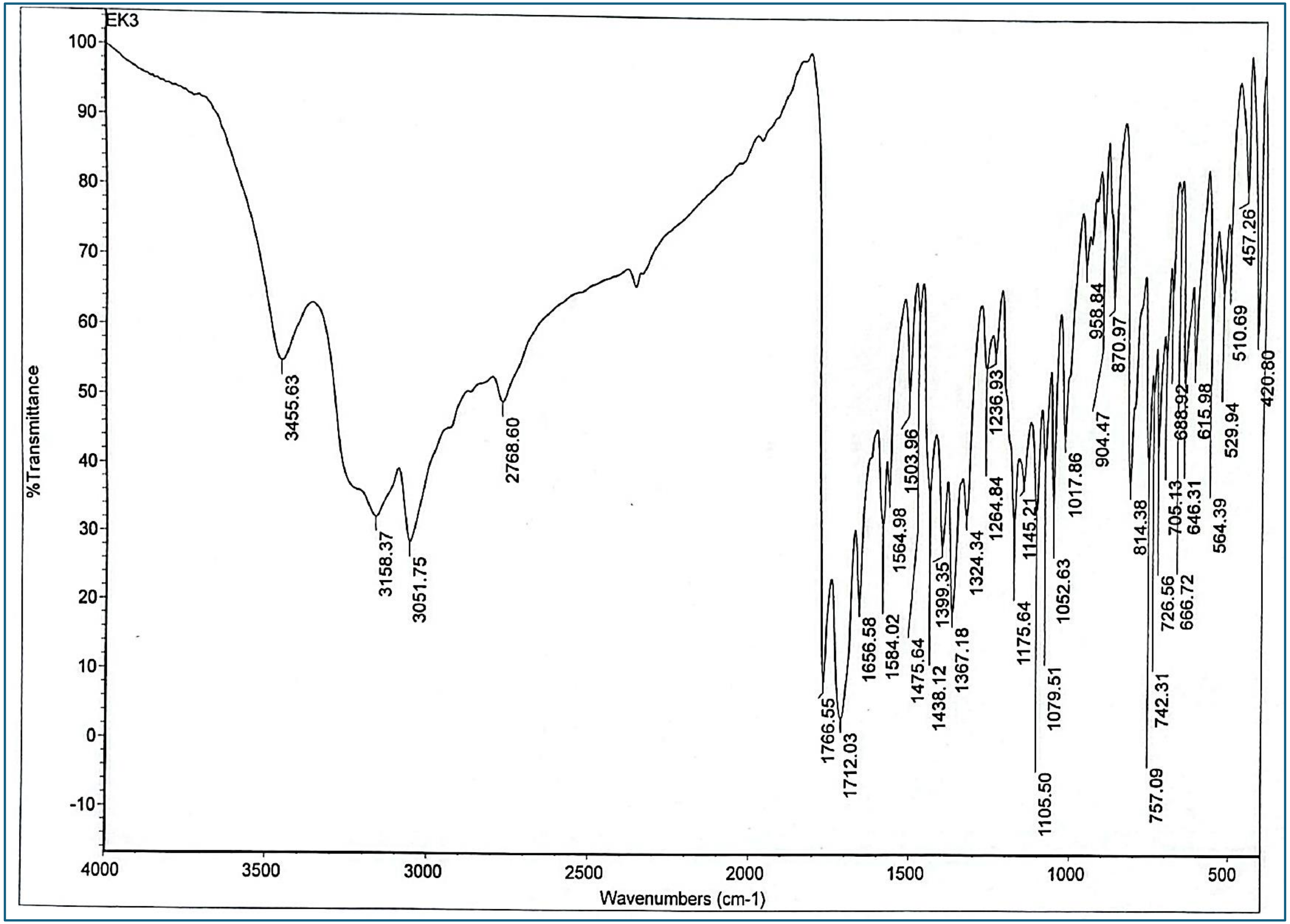

IR spectrum of compound 8

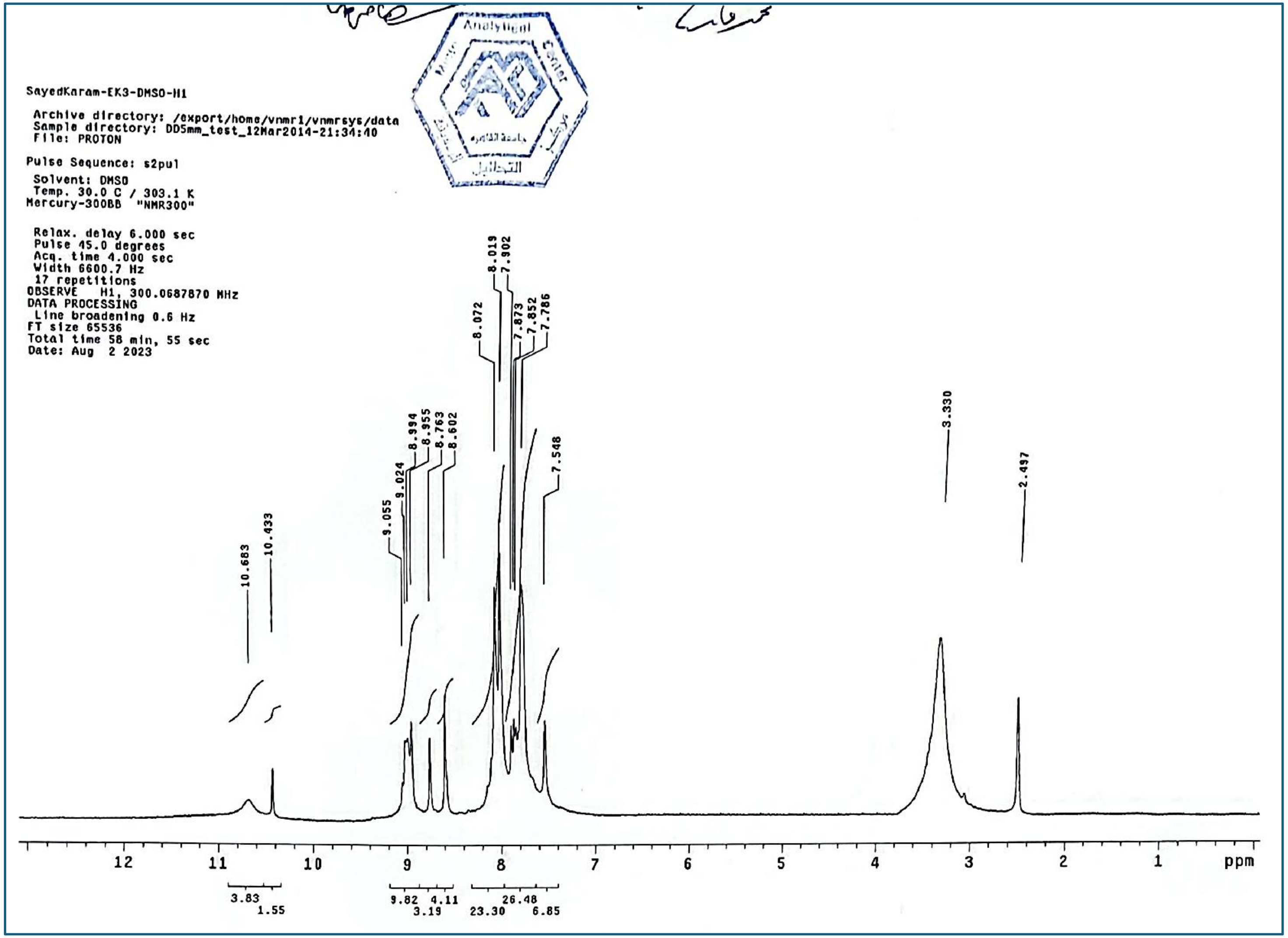

^{1}H NMR spectrum (DMSO-d_6) of compound 8

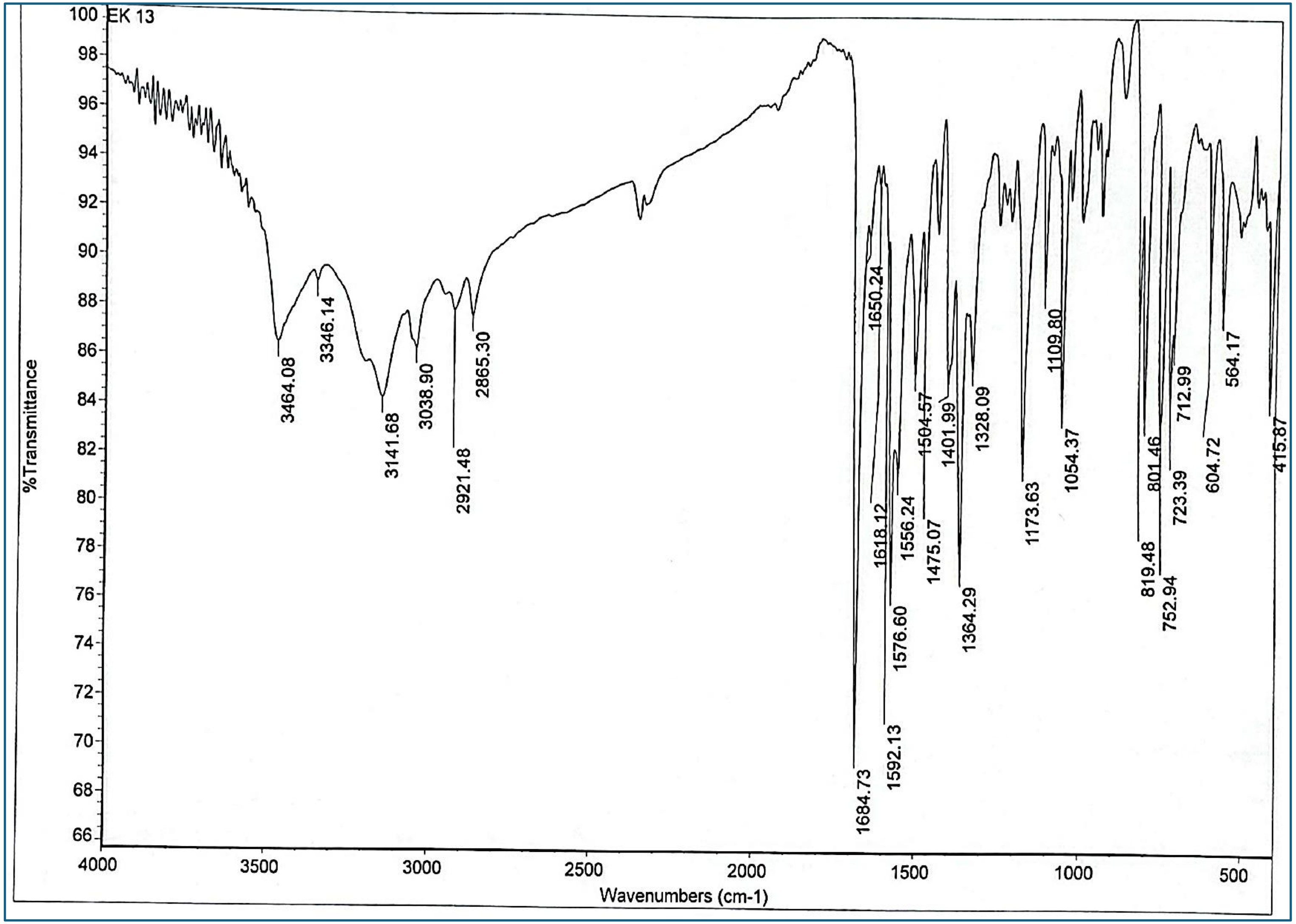

IR spectrum of compound 9

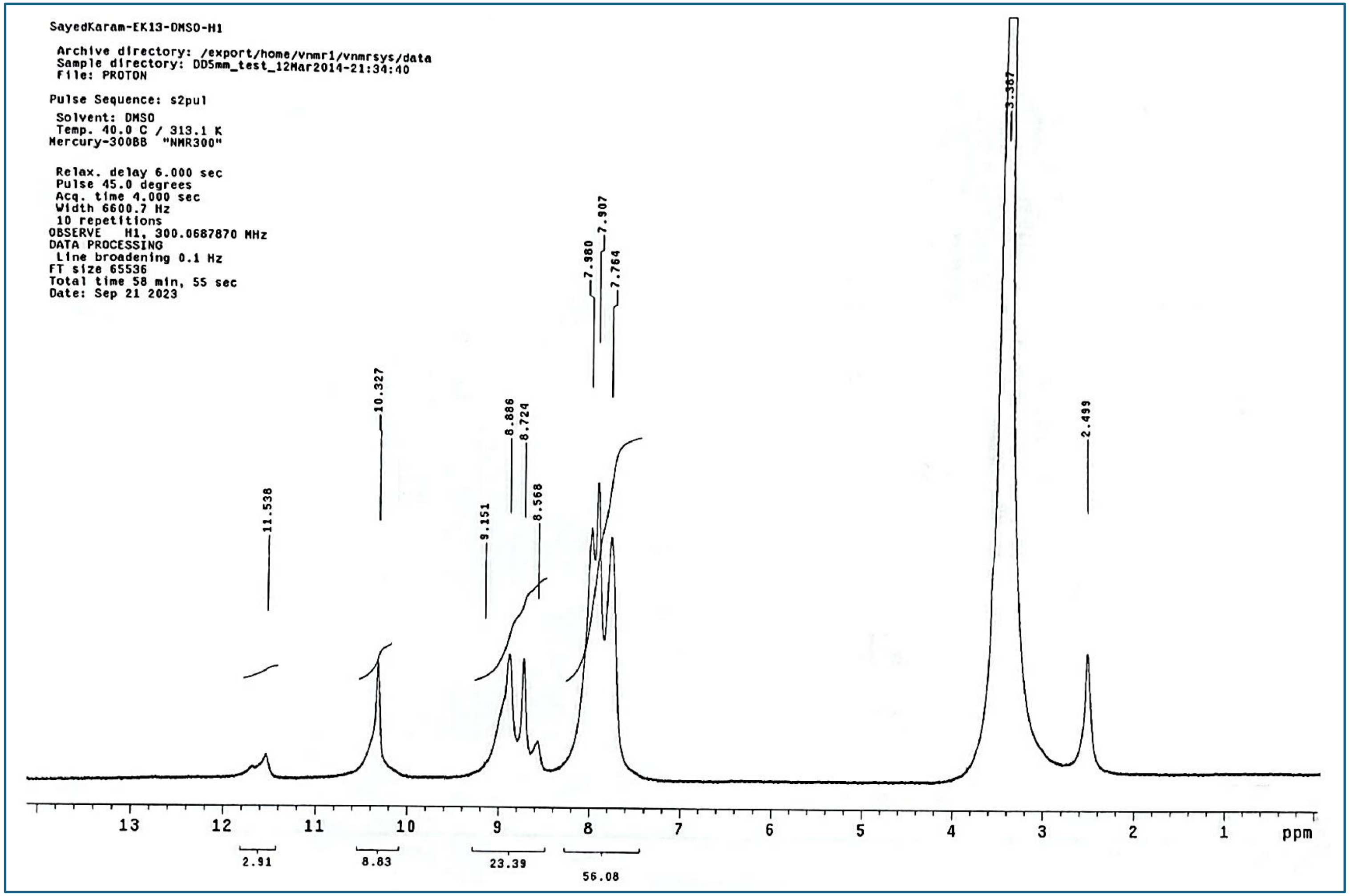

^{1}H NMR spectrum (DMSO-d_6) of compound 9

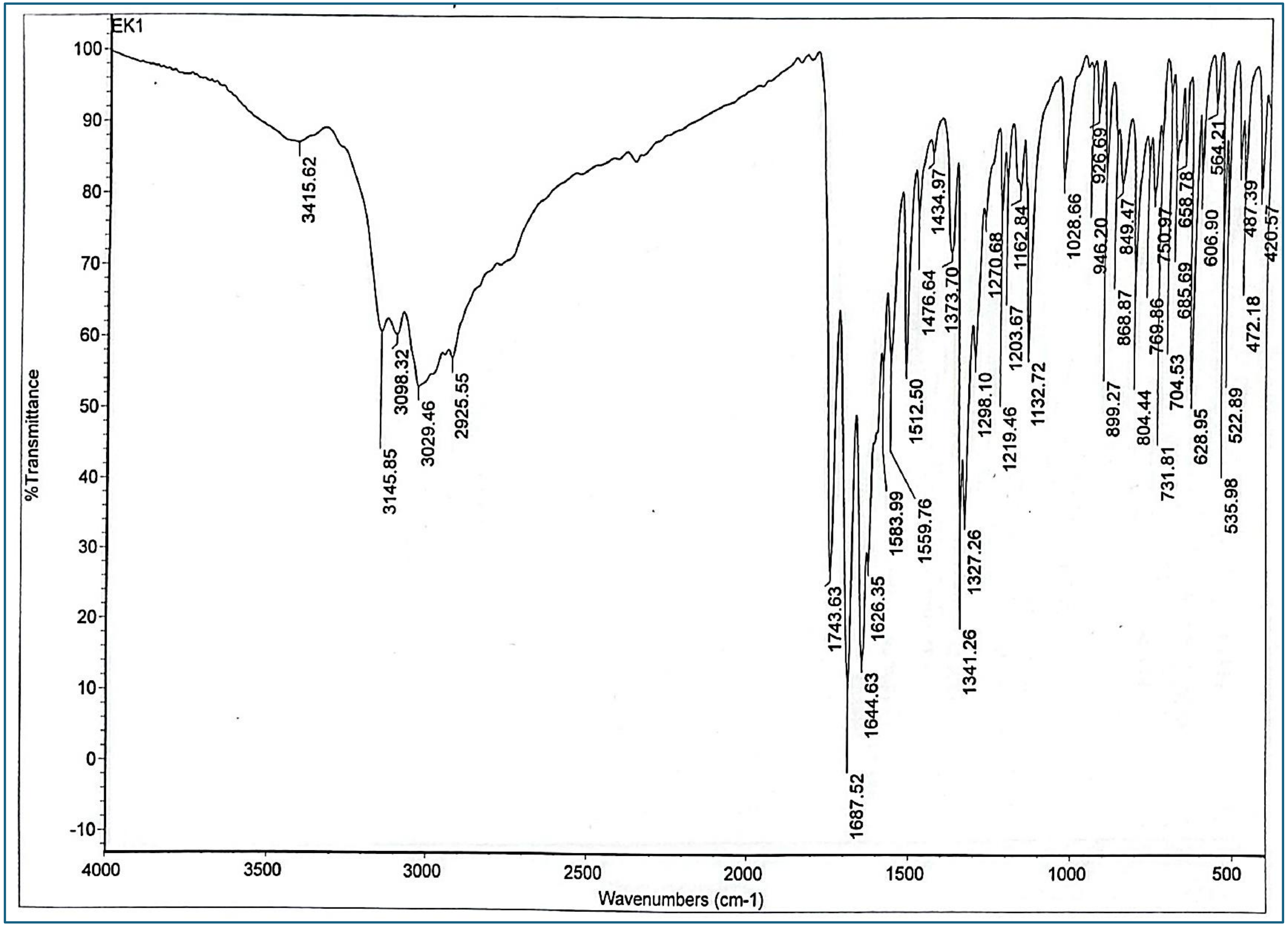

IR spectrum of compound 10

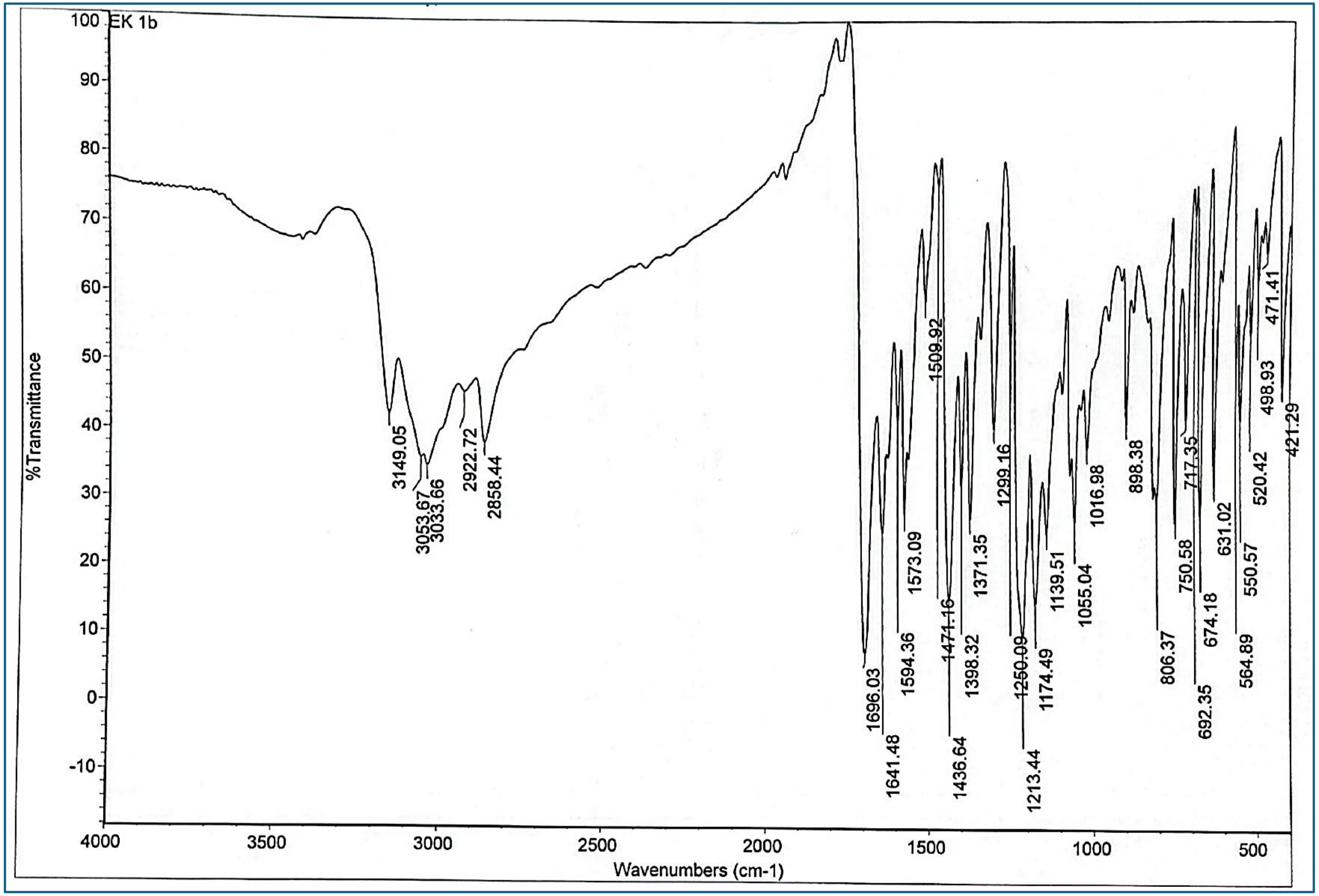

^{1}H NMR spectrum (DMSO-d_6) of compound 10

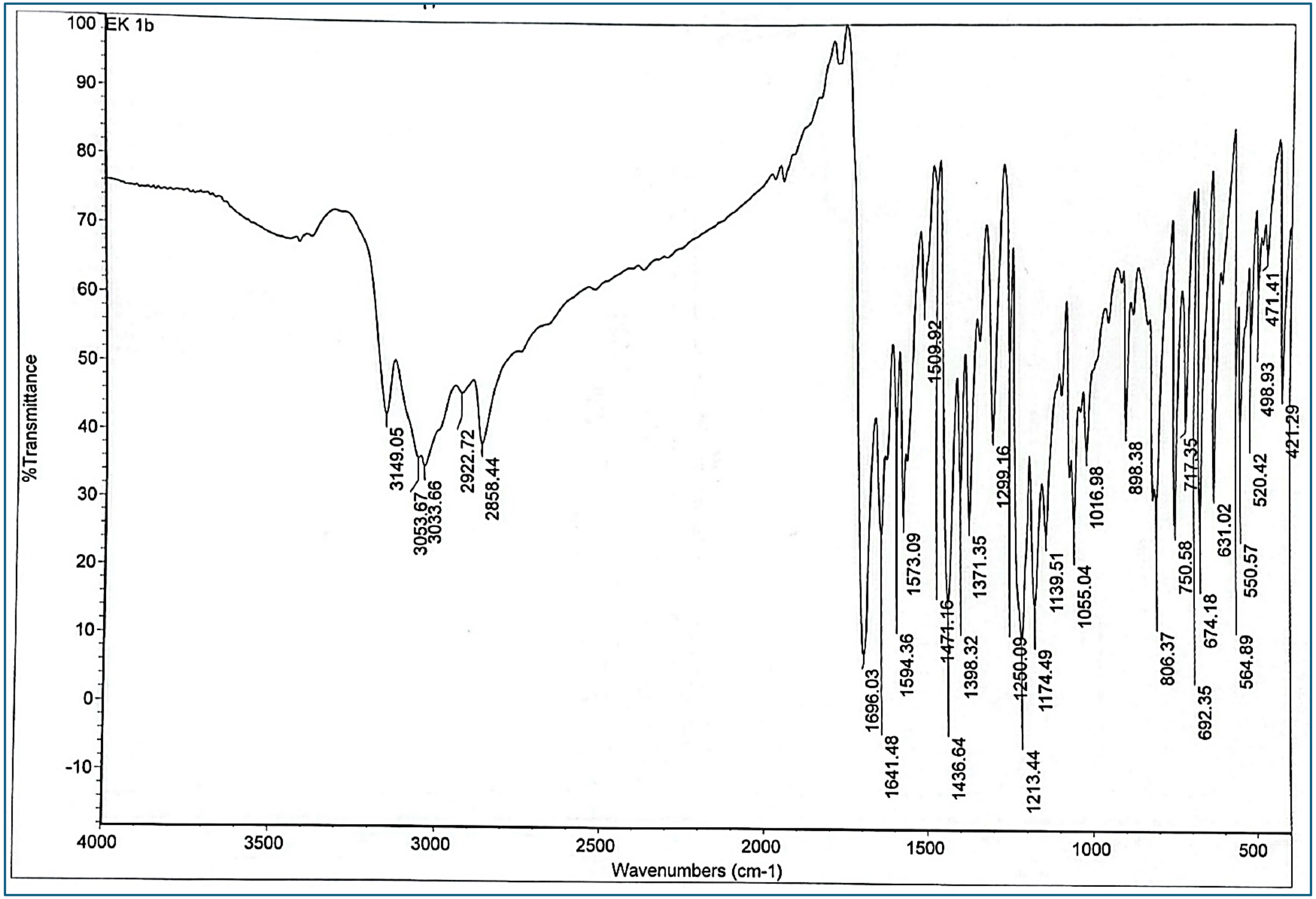

IR spectrum of compound 11

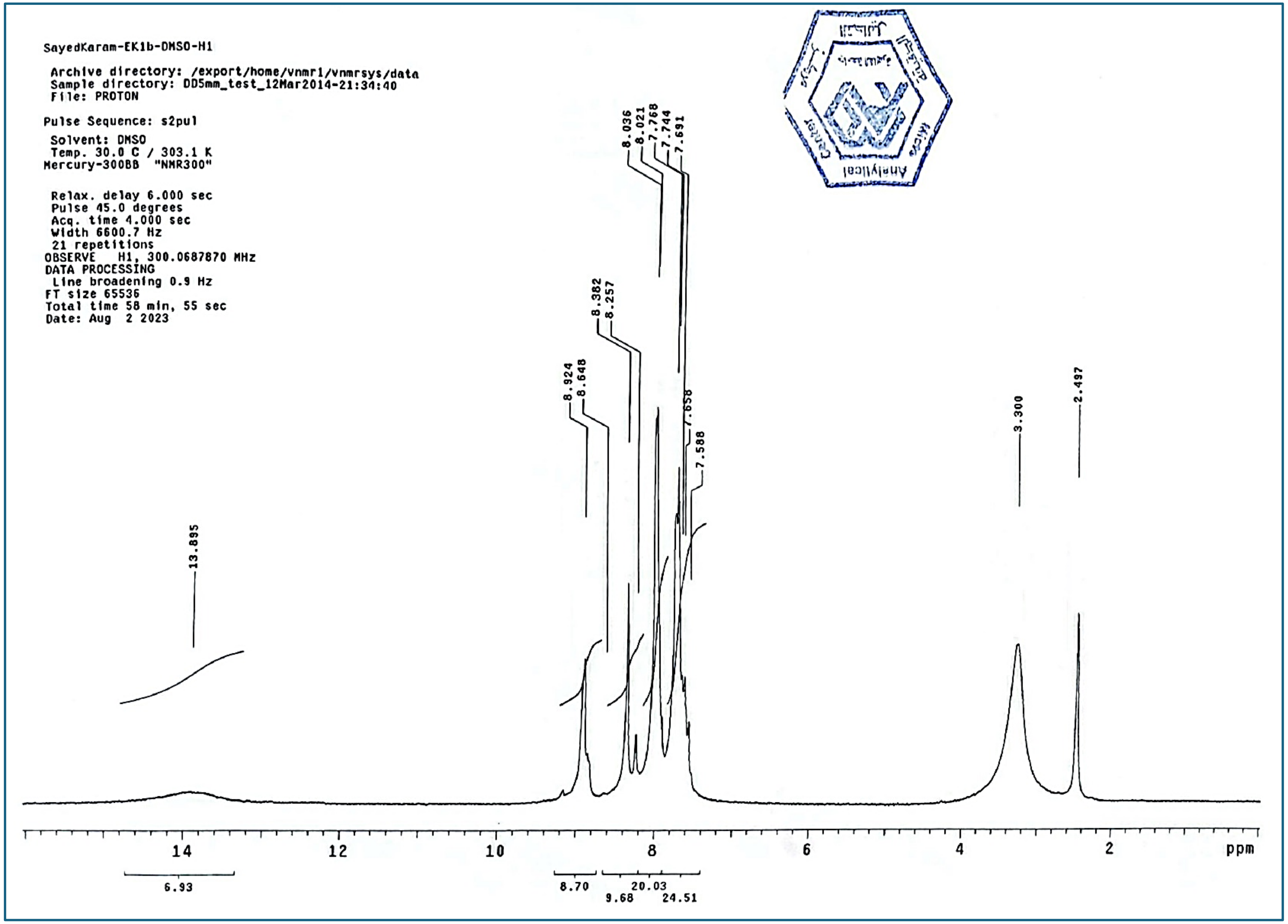

^{1}H NMR spectrum (DMSO-d_6) of compound 11

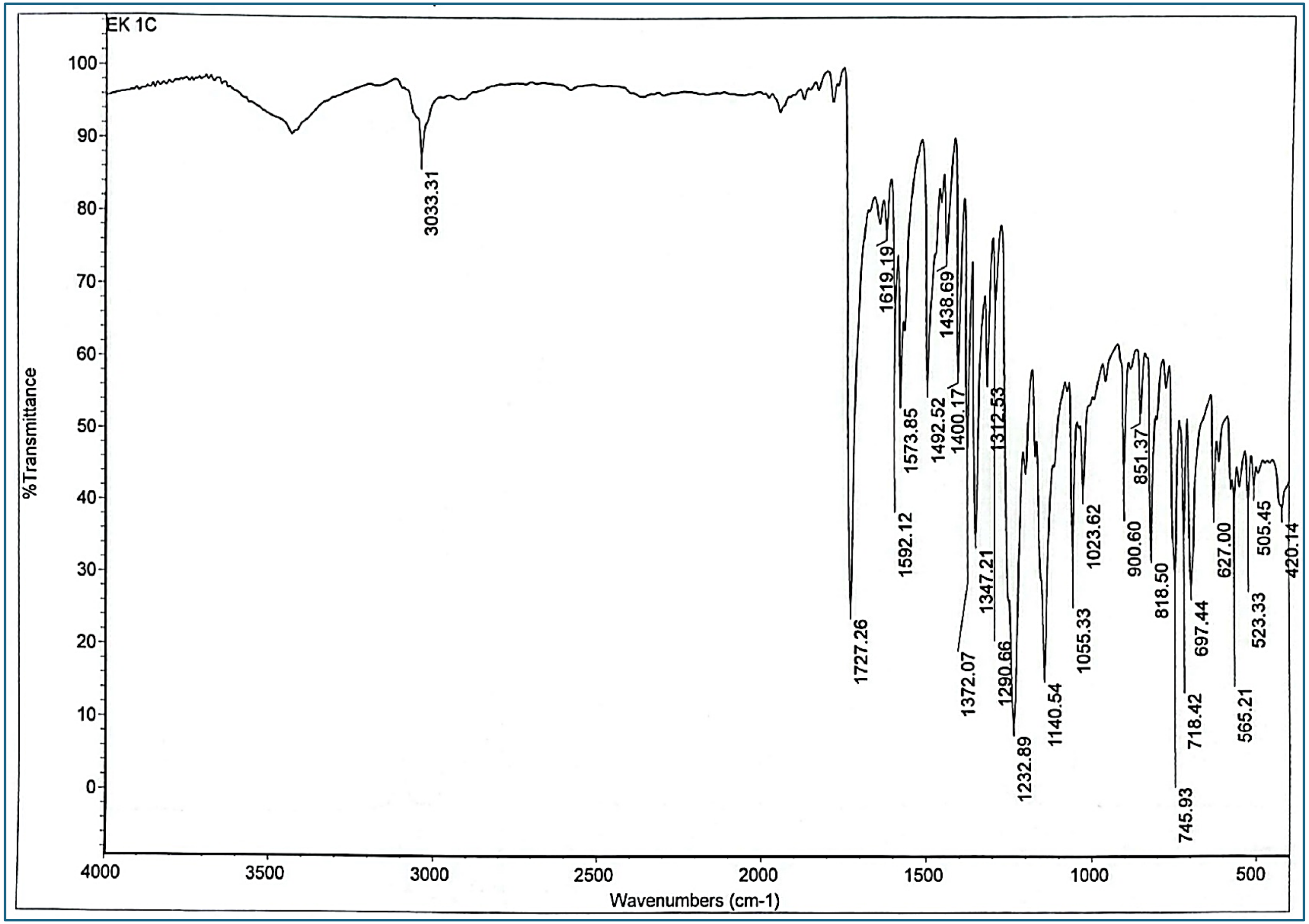

IR spectrum of compound 12

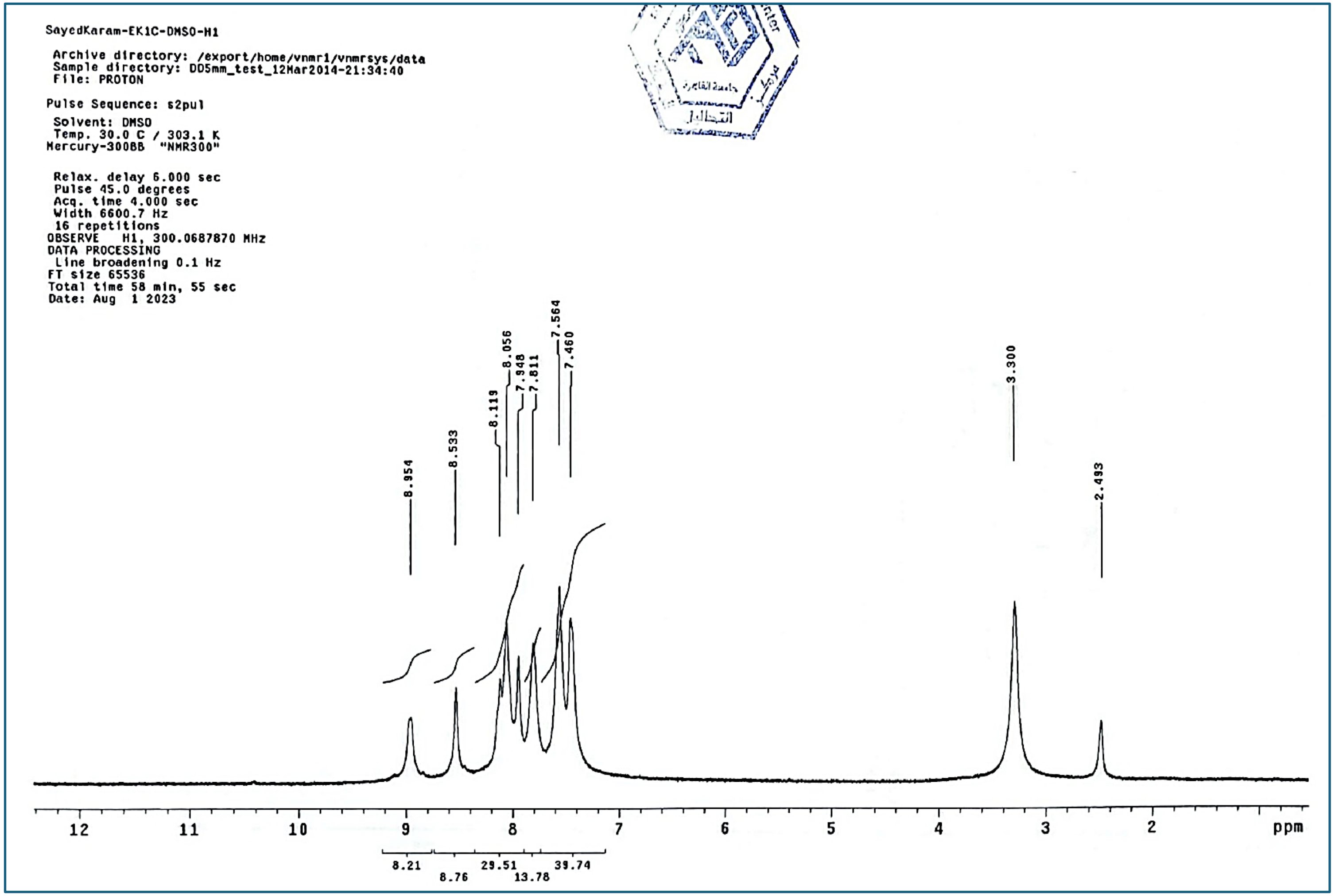

^{1}H NMR spectrum (DMSO-d_6) of compound 12

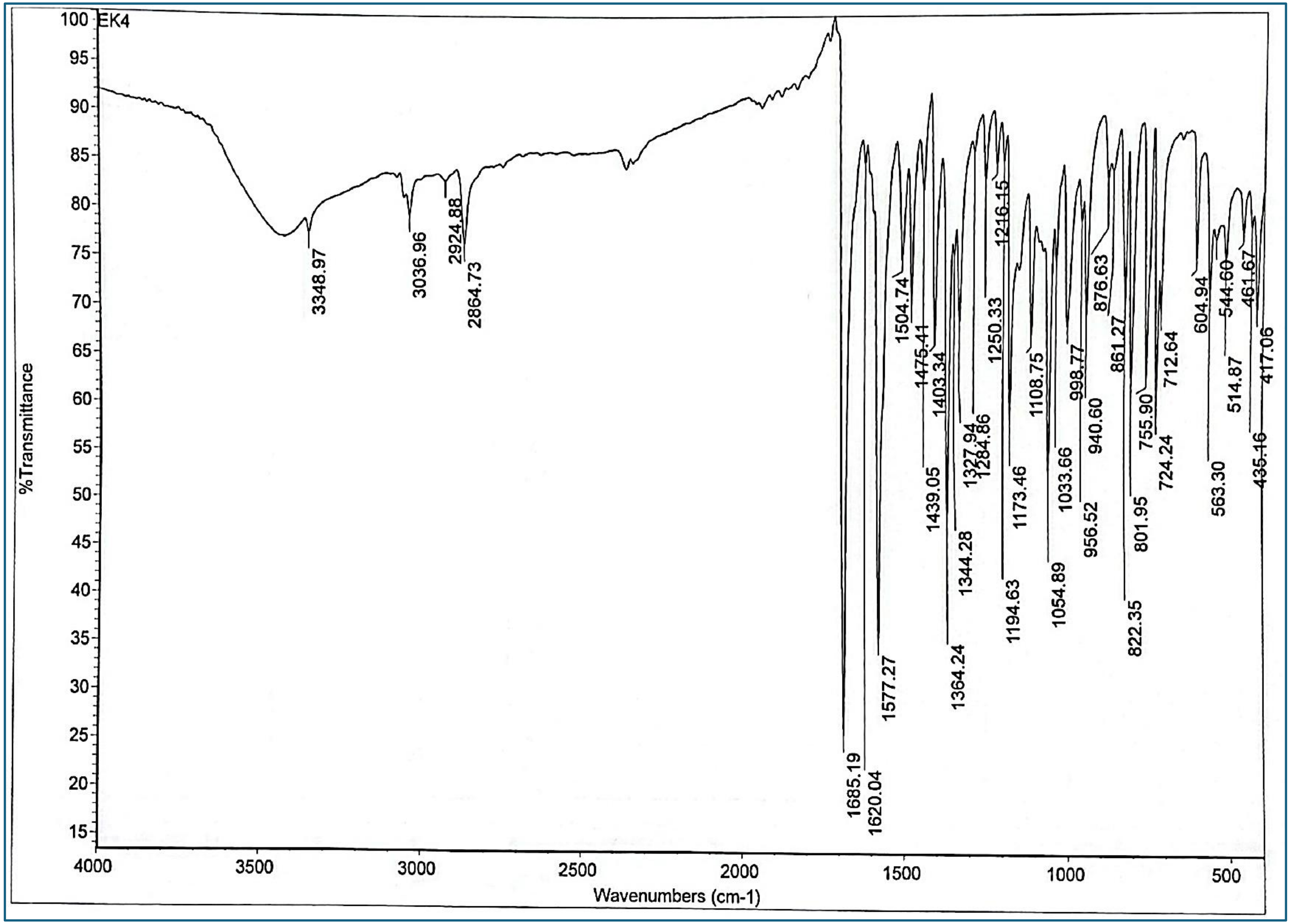

IR spectrum of compound 14

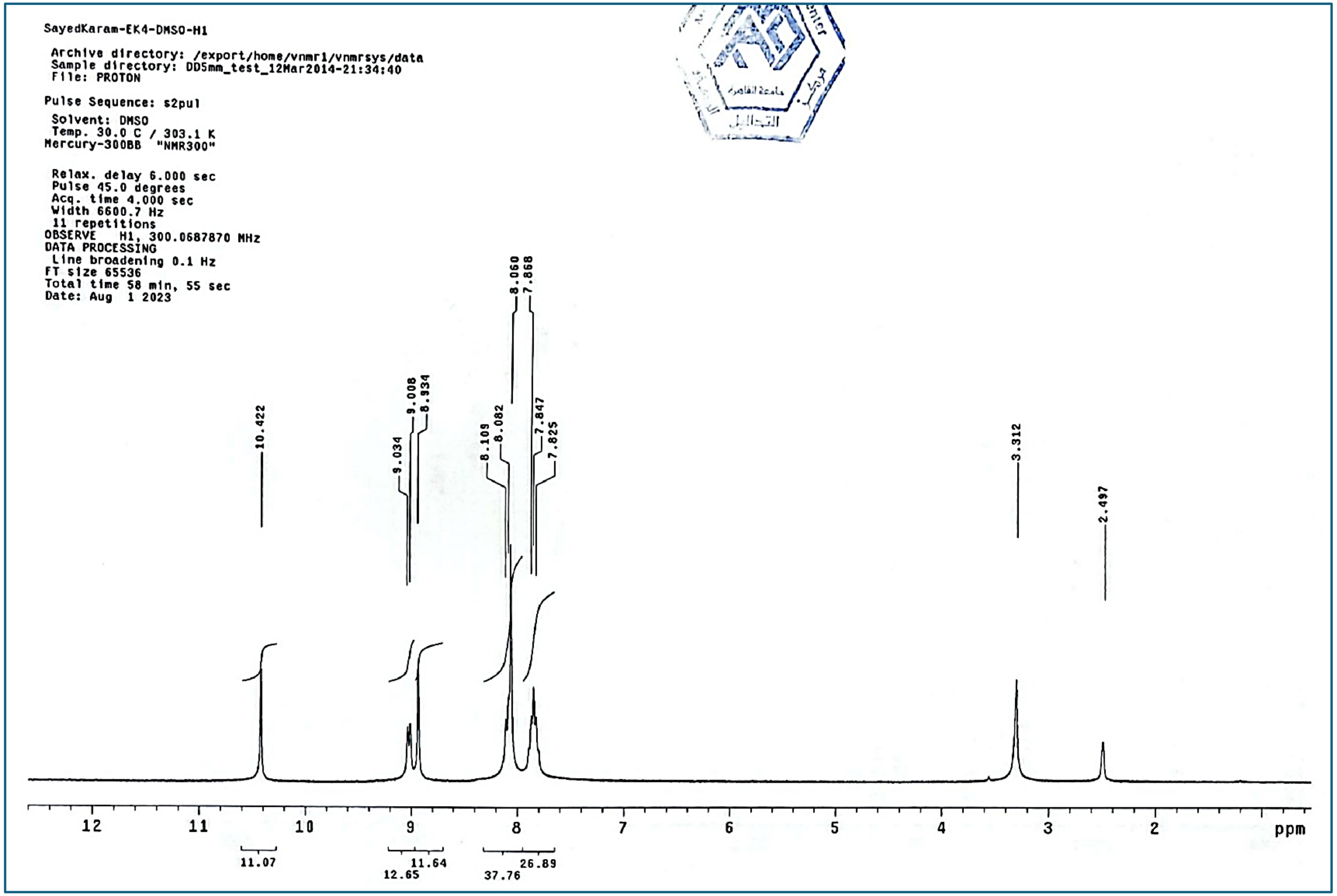

^{1}H NMR spectrum (DMSO-d_6) of compound 14

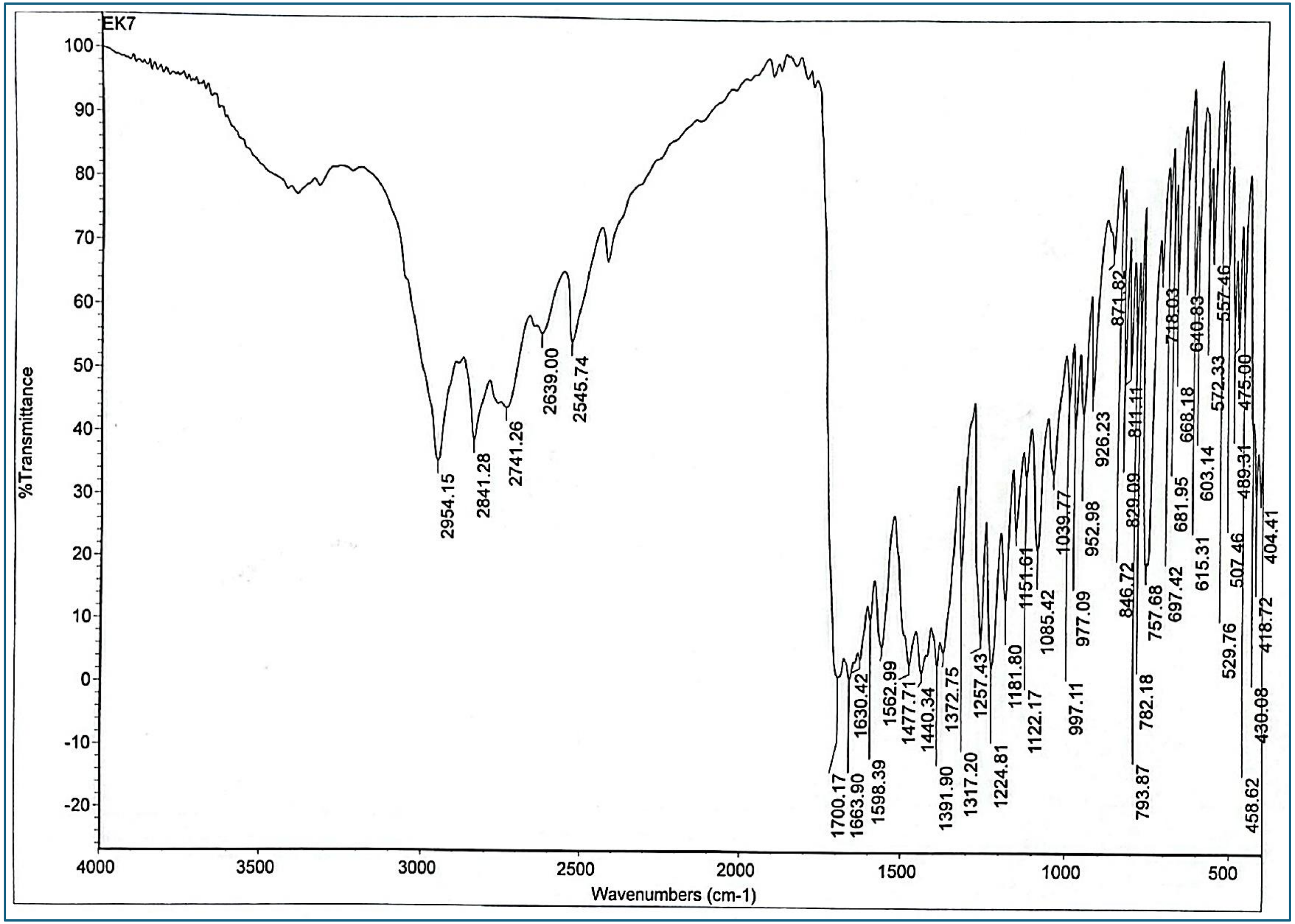

IR spectrum of compound 16

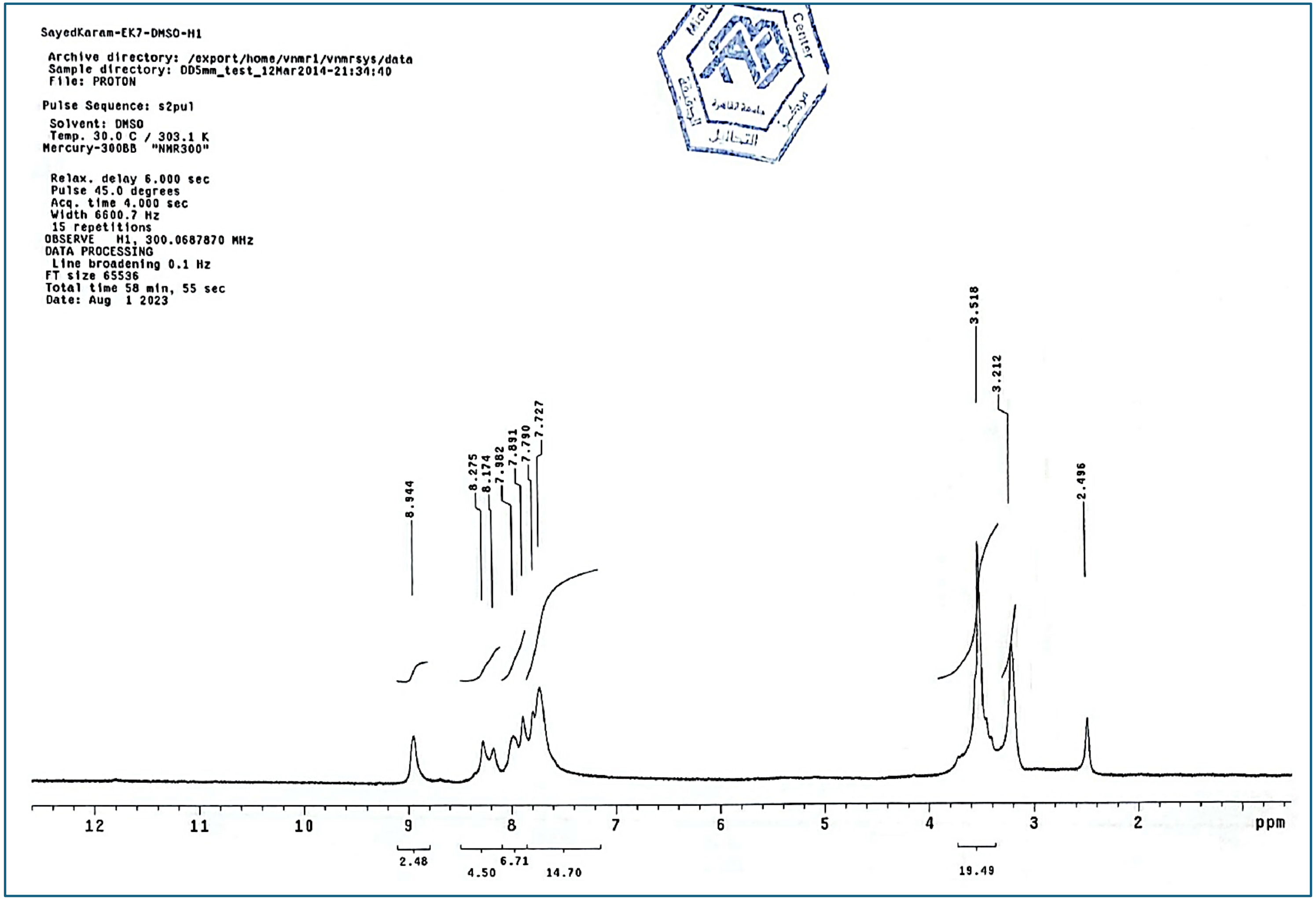

^{1}H NMR spectrum (DMSO-d_6) of compound 16

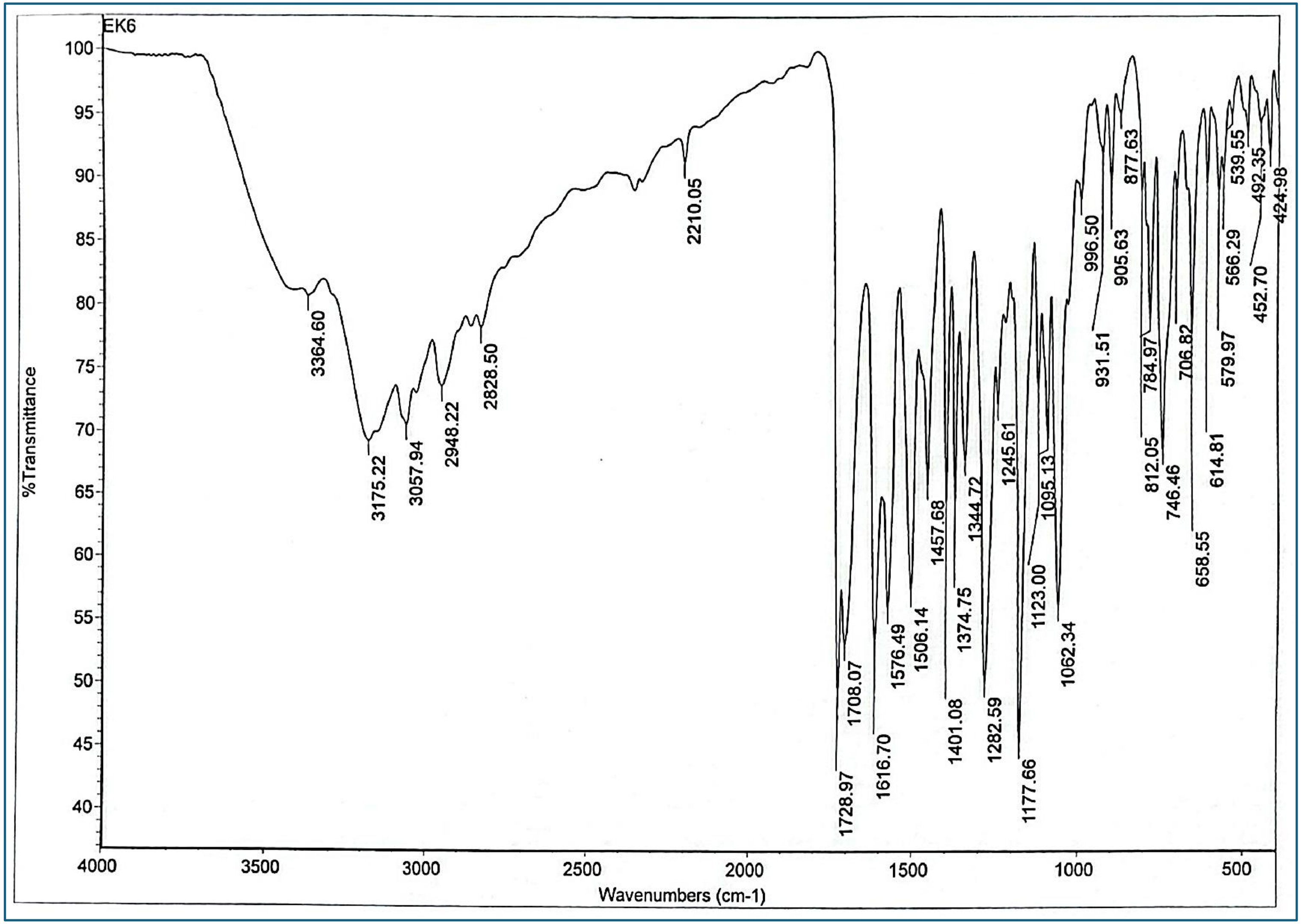

IR spectrum of compound 18

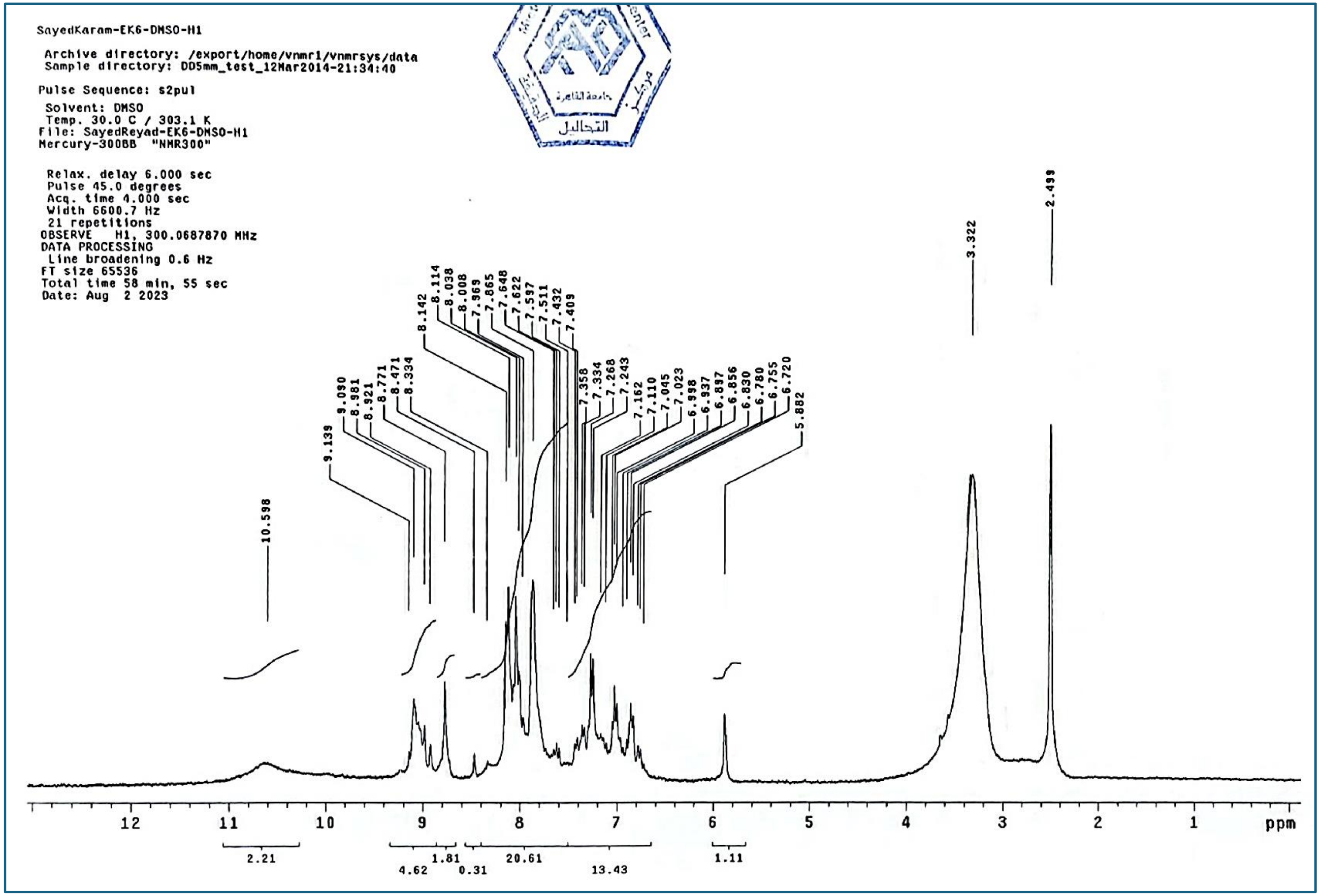

^{1}H NMR spectrum (DMSO-d_6) of compound 18
